# Optimal Sensor Placement for Multiple Target Positioning with Range-Only Measurements in Two-Dimensional Scenarios

**DOI:** 10.3390/s130810674

**Published:** 2013-08-16

**Authors:** David Moreno-Salinas, Antonio M. Pascoal, Joaquin Aranda

**Affiliations:** 1 Department of Computer Science and Automatic Control, National Distance Education University (UNED), Juan del Rosal 16, Madrid 28040, Spain; E-Mail: jaranda@dia.uned.es; 2 Institute for Systems and Robotics (ISR), Instituto Superior Tecnico (IST), University of Lisboa, Av. Rovisco Pais 1, Lisbon 1049-001, Portugal; E-Mail: antonio@isr.ist.utl.pt

**Keywords:** position estimation, positioning systems, estimation theory, localization, information analysis, optimization, autonomous vehicles, sensor networks

## Abstract

The problem of determining the optimal geometric configuration of a sensor network that will maximize the range-related information available for multiple target positioning is of key importance in a multitude of application scenarios. In this paper, a set of sensors that measures the distances between the targets and each of the receivers is considered, assuming that the range measurements are corrupted by white Gaussian noise, in order to search for the formation that maximizes the accuracy of the target estimates. Using tools from estimation theory and convex optimization, the problem is converted into that of maximizing, by proper choice of the sensor positions, a convex combination of the logarithms of the determinants of the Fisher Information Matrices corresponding to each of the targets in order to determine the sensor configuration that yields the minimum possible covariance of any unbiased target estimator. Analytical and numerical solutions are well defined and it is shown that the optimal configuration of the sensors depends explicitly on the constraints imposed on the sensor configuration, the target positions, and the probabilistic distributions that define the prior uncertainty in each of the target positions. Simulation examples illustrate the key results derived.

## 1. Introduction

Autonomous vehicles and sensor networks have become ubiquitous and are key tools in the robotics field, due to the versatility and flexibility that they show in a number of scenarios [1], their relatively low cost and the fact that their use can avoid placing human lives at risk [2]. Central to the operation of this kind of vehicles is the availability of adequate positioning systems to localize one or more vehicles simultaneously with good accuracy. In this sense, Global Positioning Systems (GPS) are commonly used for localization in a wide number of application scenarios. The advantages of a GPS system are its wide area coverage, the capability of providing navigation data seamlessly to multiple vehicles, relatively low power requirements, the miniaturization of receivers and being environmentally friendly in the sense that its signals do not interfere significantly with the ecosystem. However, there are multiple scenarios in which GPS systems are useless, as for example, indoor, caves, urban scenarios with many surrounding buildings, space or underwater.

From the above, it is clear that the problem of source localization in those areas in which the common GPS systems are useless has become increasingly important in recent years. The localization of a source (or sources) is done through a set of signals acquired by a conveniently designed sensor array. The aim of the work at hand is to determine the sensor positions of the array for which the information obtained about the source or sources is maximized, that is, the sensor placement for which the positioning accuracy is the largest possible for each of the targets involved in the task. The source positions are then defined with the information received by the sensor nodes through a convenient algorithm based on the nature of the measurements. This paper is focused on range-only measurements.

Existent localization techniques depend on the information that is available for the sensor network to define the range distances, and this information may be of a different nature, for example, power level information that consists in measuring the power level of a signal sent from a source to a sensor, known as Received Signal Strength (RSS) [3,4], the Time Difference of Arrival (TDOA) and the Time of Arrival (TOA) of the signals sent between targets and sensors [5–9], or the Angle of Arrival (AOA) of the corresponding signal [10,11]. These techniques can be implemented using radio frequency (RF) systems [12,13] or acoustic systems [14], for example, and require an accurate knowledge of the sensor positions (usually known as anchors), since any error on these positions is directly translated to errors on the source estimated position. In this work, it is considered that the range measurements are computed with any of the above techniques, and then, the optimal configuration (formation) of a sensor network that will, in a well-defined sense, maximize the range-related information available for target positioning is derived. To this effect, it is assumed that the range measurements are corrupted by white Gaussian noise, and the computation of the target position may be done by resorting to some trilateration algorithm; see [8,15,16] and the references therein.

Interesting results in the area go back to the work of [17], where the Cramer-Rao Bound is used as an indicator of the accuracy of source position estimation and a simple geometric interpretation of this bound is offered. In the same reference, the authors describe a solution to the problem of finding the sensor arrangements that minimize the bound, subject to geometric constraints. In [18], the problem of target positioning in two-dimensional (2D) scenarios is examined. The author shows explicitly what is the lowest possible geometric dilution of precision (GDOP) attainable using range or pseudo-range measurements to *N* optimally located points and determines the corresponding regular polygon-like sensor configuration. In [19], the authors study optimal sensor placement and motion coordination strategies for mobile sensor networks. For a target tracking application with range sensors, they investigate the determinant of the Fisher Information Matrix (FIM) and compute it in the 2D and 3D cases. They further characterize the global minimum in the 2D case. In [20], an iterative algorithm that places a number of sensors, so as to minimize the position estimation error bound, is developed, yielding configurations for the optimal formation subject to several complex constraints. In [10] and [9], the authors characterize the relative sensor-target geometry for positioning problems that exploit bearing-only (AOA), time-of-arrival (TOA) and time-difference-of-arrival (TDOA) strategies in 2D. Finally, in [21], the authors address the problem of localizing a source in 2D from noisy time-of-arrival measurements by seeking an extreme of the FIM for truncated, radially-symmetric source distributions that characterize prior uncertainty in the target location. Similar conclusions are found in other interesting references, such as [14, 22–25], where optimality conditions for sensor placement are derived.

From the above, it is clear that the problem of optimal sensor placement is of great interest and importance, and moreover, it plays a key role in different application areas. For example, in [26], seismic network configurations are derived to maximize the precision with which the location of earthquakes is determined. The maximization of the logarithm of the FIM determinant is used as optimality criteria. In [27], a swarm of sensors is employed in a health monitoring system for structures, such us bridges, where the optimal placement of the sensors is defined using a swarm intelligence technique, called Particle Swarm Optimization (PSO). Other noteworthy references are [28], in which a sensor network with a large number of nodes is used for surveillance, and [29], where the problem of optimal trajectory generation is studied for a group of sensors tracking a moving target using range measurements.

Motivated by recent results in ground robotics for single target positioning, this paper tackles the *multiple target positioning problem*, based on target-sensor range measurements only. Given a target positioning problem, the optimal sensor configuration can be ascertained by examining the corresponding Cramer-Rao Bound (CRB) or Fisher Information Matrix (FIM). See [30] for a lucid presentation of this subject in the context of estimation theory. In this sense, the expression of the optimal Fisher Information Matrix that provides the maximum possible information about each target is defined, and from its analytical form, the optimal sensor configurations are derived analytically, too. Clearly, there may be tradeoffs involved in the precision with which each of the targets can be localized, depending on the mission at hand; to study them, techniques that borrow from estimation theory and convex optimization are used. For the latter, the reader is referred to [31–34]. Thus, a powerful tool is obtained to determine the sensor configuration that yields, if possible, the maximum possible accuracy with which the position of the different targets can be estimated.

It is important to remark that for the optimization problem, the logarithms of the determinants of the FIMs are used. This makes the functions to be maximized jointly convex in the search parameter space. For a discussion of the convexity of the functions adopted, see, for example, [34], Chapter 3, and the work in [35] on the *D-optimality* criterion.

For a multi-target localization problem, the optimal geometry of the sensor configuration depends strongly on the constraints imposed by the task itself (e.g., maximum number and type of sensors that can be used), the environment (e.g., ambient noise), the number of targets and their configuration, and the possibly different probability distribution functions that describe the prior uncertainty of each target position. An inadequate sensor configuration may yield large localization errors for some of the targets. Even though the problem of optimal sensor placement for range-based localization is of great importance, not many results are available on this topic yet; even more, the results are only for single target positioning. Some notable exceptions include the work in [36], which, in spite of dealing with the problem of single target positioning, it addresses explicitly the design of sensor networks for the maximization of the accuracy with which a target moving along a preplanned trajectory can be positioned; moreover, uncertainty in the target position along the trajectory is considered. This can be seen as a particular case of the multi-target problem. An incremental optimization algorithm is defined to increase the likelihood of the vehicle following its intended trajectory. Similarly in [20], the authors determine numerical optimal solutions in two-dimensional constrained scenarios with an iterative optimization algorithm by minimizing the Position Error Bound (PEB) associated with each of the targets. Another interesting work is [2], in which the problem of detecting and locating subsurface objects by using a maneuvering array that receives scattered seismic surface waves is considered. The goal is to minimize the number of distinct measurements (array movements) needed to localize objects, such as buried landmines, while maximizing the determinant of the FIM. The scenario in which two targets must be localized is studied. In [37], the problem of large-scale optimal sensor management for multi-target tracking with bearings measurements is studied using optimization techniques. Finally, in [38], the computation of numerically optimal solutions for sensor placement is offered for the case of multiple underwater target positioning by a surface sensor network, showing several optimal configurations for different scenarios.

In this paper, in striking contrast to what is customary in the literature, the problem of optimal sensor placement for multi-target localization for an arbitrary number of targets is studied. At this point, it is important to point out that following what is commonly reported in the literature, the work starts by addressing the problem of optimal sensor placement given assumed positions for the targets. It may be argued that this assumption defeats the purpose of devising a method to compute the target positions, for the latter are known in advance. The rationale for the problem at hand stems from the need to first fully understand the simpler situation, where the positions of the targets are known, and to characterize, in a rigorous manner, the types of solutions obtained for the optimal sensor placement problem. In a practical situation, the positions of the targets are only known with uncertainty, and this problem must be tackled directly. However, in this case, it is virtually impossible to develop a general analytical characterization of the optimal solutions, and one must resort to numerical search methods. At this stage, an in-depth understanding of the types of solutions obtained for the ideal case is of the utmost importance to compute an initial guess for the optimal sensor placement algorithm adopted. These issues are rarely discussed in the literature, a notable exception being [21]. The organization of the paper reflects this circle of ideas in that it effectively establishes the core theoretical tools to address and solve the case when there is uncertainty in the position of the targets.

The key contributions of the present paper are threefold: (i) the optimality conditions for single target positioning are derived and characterized analytically in a simple and fast manner, and the maximum accuracy that can be obtained in the localization of a single target working in isolation is well defined; (ii) the optimality conditions that a sensor network must satisfy in order to provide the maximum possible accuracy for multiple targets are analytically determined, and several examples of analytical solutions are offered to illustrate the potential of the methodology developed; and (iii) concepts and techniques from estimation theory and convex optimization are fully exploited to obtain numerical solutions to the optimal sensor configuration problem for multiple targets when the target positions are described by probabilistic distribution functions. This allows one to capture the important fact that the target positions are only known with uncertainty

The paper is organized as follows. Section 2 defines the set-up for single target positioning, and the FIM is properly defined. The optimality conditions for the single target positioning problem are derived in Section 3, and some simulation examples are shown. Section 4 defines the problem formulation and the set-up for the multiple target positioning task. The analytical solution for the two target positioning problem is given in Section 5, and in Section 6, analytical and numerical solutions are defined for the problem of localizing an arbitrary number of targets. In Section 7, the maximization of the average value of the logarithms of the FIM determinants is studied when a sensor network surveys a certain working area or when there is uncertainty in the *a priori* knowledge about the target positions. Finally, Section 8, contains the conclusions and describes issues that warrant further research.

## 2. Fisher Information Matrix with Range-Only Measurements

**Figure 1.**
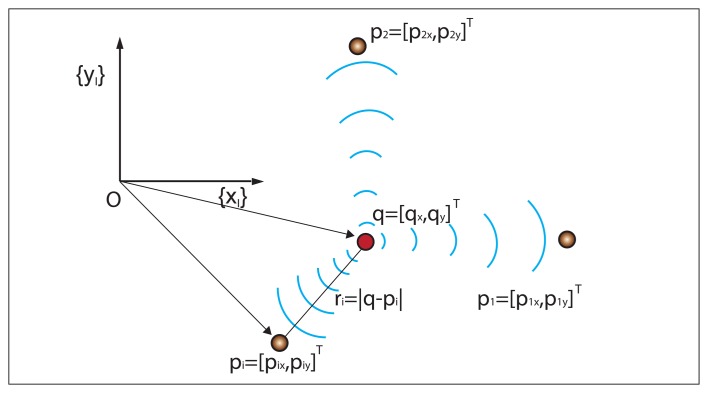
Target localization problem set-up.

Let {*I*} be an inertial reference frame with unit axis, {*x_I_*}, {*y_I_*}, and let *q* = [*q_x_*, *q_y_*]*^T^* be the position of a target to be positioned in {*I*}. Further denote by *p_i_* = [*p_ix_*, *p_iy_*]*^T^*; *i* = 1, 2,.., *n* the position of the *i* – *th* ranging sensor, also in {*I*}. Let *r_i_*(*q*) = |*q* – *p_i_*| (abbreviated *r_i_*) be the distance (range) between the target, *q*, and the *i* – *th* sensor, where | · | denotes the Euclidean norm. The variables and the set-up that will be used are illustrated in Figure 1 for the case of one target and three sensors.

Denote by *z_i_* the measurement of the actual range, *r_i_*(*q*), corrupted by additive noise, *ω_i_*. With the above notation, the measurement model adopted is given by:

(1) $$z_{i}=\left|q-p_{i}\right|+\omega_{i}=r_{i}(q)+\omega_{i}$$

where *r*(*q*) = [*r*_1_(*q*), ⋯, *r_n_*(*q*)]*^T^* is the vector of actual ranges, and *ω* = [*ω*_1_ ⋯ *ω_n_*]*^T^* is measurement noise, assuming that all noise sources, *ω_i_*, are independent and are zero mean Gaussian processes, *N*(0, Σ), with Σ = *σ*^2^ · *I*, where *I* is the identity matrix.

In the set-up adopted, the measurement noise model is considered to be distance-independent, in line with common assumptions reported in the literature for theoretical research and systems implementation. Some of the examples that support the model adopted (that is, constant covariance of the measurement errors) include the work of [39] on the problem of range-only vehicle localization and that of [40] on the challenging problem of optimal sensor-target localization geometries. Other interesting references where the distance-independent assumption is adopted are [15,41,42] or [29].

In general, the problem of range measurement noise modeling is not trivial. For example, for marine acoustic sensors, range measurements may be strongly affected by multipath effects, Doppler effects, energy attenuation and even uncertainty in the speed of propagation of sound in the physical medium. This uncertainty in the speed of propagation can be reduced by measuring the speed of sound at the operation site. Energy attenuation, with its impact on signal-to-noise (SN) ratio, as well as signal distortion, due to the characteristics of the physical medium, can be dealt with very effectively using acoustic ranging devices that build on spread spectrum techniques and employ cross-correlation techniques for the detection of incoming waves and, therefore, for the measurement of their times of arrival. As a consequence, from short to medium ranges, as long as the SN ratio does not cross a device-dependent limit, the statistics of the measurement errors can be taken as approximately constant. Identical considerations apply to modern devices that can compensate for Doppler effects. The problems caused by multipath effects and/or ray bending, due to the geometry and characteristics of the channel, are of an entirely different breed and will not be considered in the present work. Moreover, it is clearly stated in [43] that for direct path measurements, the range measurement errors can be assumed to be Gaussian with constant covariance, *i.e.*, the non-Gaussian characteristic only arises in the presence of outliers and multipath effects. These comments are extensive to range measurements obtained with different types of sensors. In view of the above considerations, the model adopted in this paper for range measurement errors captures a large number of practical scenarios and allows for a rigorous characterization of the theoretical solutions for optimal sensor placement.

In what follows, it is assumed that the reader is familiar with the concepts of Cramer-Rao Lower Bound (CRLB) and Fisher Information Matrix (FIM); see, for example, [30]. Stated in simple terms, the FIM captures the amount of information that measured data provides about an unknown parameter (or vector of parameters) to be estimated. Under known assumptions, the FIM is the inverse of the Cramer-Rao Bound matrix (abbreviated CRB), which lower bounds the covariance of the estimation error that can possibly be obtained with any unbiased estimator. Thus, “minimizing the CRB” may yield (by proper estimator selection) a decrease of uncertainty in the parameter estimation. Formally, let *q̂*(*z*) be any unbiased estimator of *q*, that is, a mapping, *q̂*: ℜ*^n^*→ℜ^2^, between the observations, *z*, and the target position space, such that *E*{*q̂*} = *q* for all *q* ∈ ℜ^2^, where *E*{·} denotes the average operator. Let 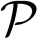
*_q_*(*z*) be the likelihood function that defines the probability of obtaining the observation, *z*, given that the true target position is *q*. It is well known that under some regularity conditions on 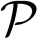
*_q_*(*z*), the following inequality holds:

(2) $$\text{Cov}\{\hat{q}\}\geq \text{FIM}{\left(q\right)}^{-1}=\text{CRB}(q)$$

where:

(3) $$\text{Cov}\{\hat{q}\}=E\{(\hat{q}-q){\left(\hat{q}-q\right)}^{T}\}$$

*FIM* (*q*) (often abbreviated simply as FIM) is the Fisher Information Matrix, defined as:

(4) $$\text{FIM}(q)=E\{(\nabla_{q}\log P_{q}(z)){\left(\nabla_{q}\log P_{q}(z)\right)}^{T}\}$$

and *CRB* (*q*) is the Cramer-Rao Bound matrix. In the above, ∇*_q_* log 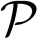
*_q_* denotes the gradient of the log of the likelihood function with respect to the unknown parameter, *q*. Taking the trace of both sides of the covariance inequality yields:

(5) $$\text{var}\left\{\hat{q}\right\}:=tr(\text{Cov}\left\{\hat{q}\right\})=tr(E\{(\hat{q}-q){\left(\hat{q}-q\right)}^{T}\})\geq tr{\left(\text{FIM}(q)\right)}^{-1}$$

which sets a lower bound on the mean-square error of any unbiased estimator.

Equipped with the above notation and tools of estimation theory, the optimal sensor placement problem is now addressed by solving a related equivalent optimization one: given the FIM for the problem at hand, maximize the logarithm of its determinant by proper choice of the sensor coordinates. This strategy for sensor placement underlies much of the previous work available in the literature; see, for example, [21,39] and the references therein. Following standard procedures, the FIM corresponding to the problem of range-based single target positioning can be computed from the likelihood function, 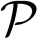
*_q_*(*z*), given by:

(6) $$P_{q}(z)=\frac{1}{{\left(2\pi \right)}^{\frac{n}{2}}{\left|\sum \right|}^{\frac{1}{2}}}\exp\left\{-\frac{1}{2}{\left(z-r(q)\right)}^{T}\sum^{-1}(z-r(q))\right\}$$

where *n* is the number of receivers, *z* = [*z*_1_, *z*_2_, …, *z_n_*]*^T^* consists of *n* measured ranges and *r*(*q*) are the actual ranges. Taking the logarithm of Equation (6), computing its derivative with respect to *q* and, then, its expected value, the FIM is defined as:

(7) $$\text{FIM}=C{\left(\delta (r)\sum \delta (r)\right)}^{-1}C^{T}$$

where $C=(q1_{n}^{T}-\bar{p})\in {ℜ}^{2xn}$, $1_{n}\in {ℜ}^{nx1}$ is a vector of 1s, and *p̅* is the vector of sensor positions, the latter being defined in ℜ^2^*^xn^*. In the above, *δ* is the operator, *diag*, which either converts a square matrix into a vector consisting of its diagonal elements or converts a vector into a square diagonal matrix whose diagonal components are the array elements. Once the FIM is defined, the Cramer-Rao Bound matrix is computed as *CRB* = *FIM*^−1^. In this context, the optimal sensor placement strategy is obtained by maximizing the logarithm of the determinant of the FIM, which must be computed explicitly. To this effect, Equation (7) is expanded to obtain:

(8) $$\text{FIM}=\frac{1}{\sigma^{2}}\sum_{i=1}^{n}\left(\begin{array}{cc} {\left(u_{ix}\right)}^{2} & \left(u_{iy})(u_{ix}\right)\\ \left(u_{ix})(u_{iy}\right) & {\left(u_{iy}\right)}^{2} \end{array}\right)$$

where:

(9) $$u_{i}={\left[u_{ix},u_{iy}\right]}^{T}={\left[\frac{\partial \left|q-p_{i}\right|}{\partial q_{x}},\frac{\partial \left|q-p_{i}\right|}{\partial q_{y}}\right]}^{T}$$

Clearly, the expression of the FIM is well defined.

As shown in Section 1, there are many references regarding the 2D target positioning problem. The optimal solutions for sensor placement given in those references are recovered in this paper using a novel methodology, and the results are then extended for the multiple target positioning problem. Furthermore, in this work, not only optimal formations are explicitly defined, the optimality conditions that the formations must satisfy in order to minimize the estimation error are well defined, and therefore, any possible optimal configuration may be defined using them. Optimal sensor placement examples are shown at the end of each section to illustrate the potential of the methodology developed.

## 3. Single Target Positioning

In this section, for the sake of completeness, the optimal sensor placement problem for single target positioning is studied. The aim of this section is to recover the results on optimal sensor placement defined in the literature, but with a novel methodology with which the optimality conditions for optimal sensor placement can be defined in a fast and simple manner. The results defined in this section will be used to check the efficacy of the sensor configurations developed for the multiple target positioning problem in the forthcoming sections.

### 3.1. Optimal Fisher Information Matrix

As stated in Section 1, the FIM captures the amount of information that measured data provide about an unknown parameter (or vector of parameters) to be estimated, and the log determinant of the FIM is used for the computation of an indicator of the performance that is achievable with a given sensor configuration. As mentioned above, let *q* = [*q_x_*, *q_y_*]*^T^* be the position of an arbitrary target, *p_i_* = [*p_ix_*, *p_iy_*]*^T^*; *i* = 1, 2,.., *n*, the position of the *i-th* ranging sensor, and *ω_i_* the corresponding measurement noise. Further, let *r_i_* be the actual distance between target *q* and the *i-th* sensor. For the sake of simplicity and without loss of generality, the target is considered to be placed at the origin of the inertial coordinate frame. Therefore, Equation (8) becomes:

(10) $$\text{FIM}=\frac{1}{\sigma^{2}}\sum_{i=1}^{n}\left(\begin{array}{cc} \frac{p_{ix}^{2}}{r_{i}^{2}} & \frac{p_{ix}p_{iy}}{r_{i}^{2}}\\ \frac{p_{ix}p_{iy}}{r_{i}^{2}} & \frac{p_{iy}^{2}}{r_{i}^{2}} \end{array}\right)=\frac{1}{\sigma^{2}}\sum_{i=1}^{n}\left(\begin{array}{cc} \cos^{2}\left(\alpha_{i}\right) & \cos\left(\alpha_{i}\right)\sin\left(\alpha_{i}\right)\\ \cos\left(\alpha_{i}\right)\sin\left(\alpha_{i}\right) & \sin^{2}\left(\alpha_{i}\right) \end{array}\right)$$

where *α_i_* is the angle that the *i-th* range vector forms with the {*x_I_*} axis of the inertial coordinate frame. At this point, it is convenient to introduce the vectors, X and ϒ, in ℜ*^n^* (where *n* is the number of sensors involved in the target positioning task), defined as:

(11) $$\begin{array}{l} \begin{array}{c} \text{X}=\left[\begin{array}{ccc} \cos(\alpha_{1}) & \ldots & \cos(\alpha_{n}) \end{array}\right]=\left[\begin{array}{ccc} \frac{p_{1x}}{r_{1}} & \ldots & \frac{p_{nx}}{r_{n}} \end{array}\right]\\ ϒ=\left[\begin{array}{ccc} \sin(\alpha_{1}) & \ldots & \sin(\alpha_{n}) \end{array}\right]=\left[\begin{array}{ccc} \frac{p_{1y}}{r_{1}} & \ldots & \frac{p_{ny}}{r_{n}} \end{array}\right] \end{array} \end{array}$$

As a consequence, the FIM is parametrized by two vectors in ℜ*^n^* instead of *n* vectors in ℜ^2^. It is also convenient to consider these vectors as elements of the Hilbert space, with elements in ℜ*^n^*, endowed with an inner product structure. The latter, as it is well known, allows for computation of the length of a vector and also for the angle between two vectors. The dot product between two vectors can be rewritten as the product of the norms of those vectors times the cosine of the angle between them. Simple computations allow one to rewrite Equation (10) as:

(12) $$\text{FIM}=\frac{1}{\sigma^{2}}\left(\begin{array}{cc} \text{X}\cdot \text{X} & \text{X}\cdot ϒ\\ \text{X}\cdot ϒ & ϒ\cdot ϒ \end{array}\right)=\frac{1}{\sigma^{2}}\left(\begin{array}{cc} {\left|\text{X}\right|}^{2} & \left|\text{X}\right|\left|ϒ\right|\cos(\theta )\\ \left|\text{X}\right|\left|ϒ\right|\cos(\theta ) & {\left|ϒ\right|}^{2} \end{array}\right)$$

The determinant of Equation (12) yields:

(13) $$\left|\text{FIM}\right|=\frac{1}{\sigma^{4}}{\left|\text{X}\right|}^{2}{\left|ϒ\right|}^{2}(1-\cos^{2}(\theta ))$$

where *θ* is the angle formed by vectors X and ϒ.

To determine the conditions for which *log* (|*FIM*|) is maximum (and, consequently, the optimal sensor configuration), one simply computes the derivatives of the logarithm of Equation (13) with respect to the norms of the vectors and with respect to the angle that appears explicitly in |*FIM*| and equals the derivatives to zero. Setting the derivative with respect to *θ* equal to zero yields the first necessary condition of optimality.

(14) $$\frac{\partial \log(\left|\text{FIM}\right|)}{\partial \theta }=\frac{\partial \left|\text{FIM}\right|}{\left|\text{FIM}\right|}=-\frac{2\cos(\theta )\sin(\theta )}{1-\cos^{2}(\theta )}=0$$

Clearly, sin (*θ*) = 0 provides an indetermination. From L'Hopital's rule, it is easy to check that the limit of Equation (14) tends to infinity (moreover, |*FIM*| = 0), so this solution can be discarded. Then, the only feasible solution is cos (*θ*) = 0. This solution implies that *θ* = *c* · *π*/2, where *c* is any odd natural number, and therefore, vectors X and ϒ are orthogonal. Hence, a necessary condition (to obtain the optimal sensor network that maximizes the FIM determinant) is that these two vectors must form an orthogonal system. This condition leads to a diagonal FIM.

(15) $$\text{FIM}=\frac{1}{\sigma^{2}}\left(\begin{array}{cc} {\left|\text{X}\right|}^{2} & 0\\ 0 & {\left|ϒ\right|}^{2} \end{array}\right)$$

Now, the focus is on the derivatives of the logarithm of Equation (13) with respect to the norms of the vectors. Because:

(16) $$\frac{p_{ix}^{2}}{r_{i}^{2}}+\frac{p_{iy}^{2}}{r_{i}^{2}}=1$$

it follows that:

(17) $${\left|\text{X}\right|}^{2}+{\left|ϒ\right|}^{2}=\sum_{i=1}^{n}\frac{p_{ix}^{2}}{r_{i}^{2}}+\sum_{i=1}^{n}\frac{p_{iy}^{2}}{r_{i}^{2}}=n$$

Therefore, Equation (15), together with Equation (17), can be rewritten as:

(18) $$\text{FIM}=\frac{1}{\sigma^{2}}\left(\begin{array}{cc} n-{\left|ϒ\right|}^{2} & 0\\ 0 & {\left|ϒ\right|}^{2} \end{array}\right)$$

The logarithm of the determinant of the FIM can now be written as:

(19) $$\log(\left|\text{FIM}\right|)=\log\left(\frac{1}{\sigma^{4}}{\left|ϒ\right|}^{2}(n-{\left|ϒ\right|}^{2})\right)$$

Thus, the derivative of Equation (19) with respect to the norm of the vector, ϒ, after some simplifications, yields:

(20) $$\frac{\partial \log(\left|\text{FIM}\right|)}{\partial \left|ϒ\right|}=\left|ϒ\right|(n-{\left|ϒ\right|}^{2})-{\left|ϒ\right|}^{3}=0$$

whose only valid solution is |ϒ|^2^ = *n*/2, since |ϒ| and |X| are always positive and different from zero. It is clear from Equation (17) that |X|^2^ = *n*/2, so |X|^2^ = |ϒ|^2^. Therefore, the expression of the Fisher Information Matrix that provides the maximum possible (logarithm of the) FIM determinant yields:

(21) $$FIM_{\text{opt}}=\frac{1}{\sigma^{2}}\left(\begin{array}{cc} \frac{n}{2} & 0\\ 0 & \frac{n}{2} \end{array}\right)$$

and the value of the determinant of Equation (21) is:

(22) $$\left|FIM_{\text{opt}}\right|=\frac{n^{2}}{4\sigma^{4}}$$

It is important to comment that Equation (22) provides the same optimal FIM determinant defined in [9,39], but computed in a simpler manner. Comparing the optimal FIM in Equation (21) with the generic one in Equation (8) gives an implicit characterization of the conditions that must be satisfied by the sensor network in order for it to be optimal:

(23) $$\begin{array}{l} \sum_{i=1}^{n}\frac{p_{ix}^{2}}{r_{i}^{2}}=\frac{n}{2}\\ \sum_{i=1}^{n}\frac{p_{iy}^{2}}{r_{i}^{2}}=\frac{n}{2}\\ \sum_{i=1}^{n}\frac{p_{ix}p_{iy}}{r_{i}^{2}}=0 \end{array}$$

Thus, from Equation (23), it is clear that the angles that the range vectors form between them define the optimality conditions characterizing, for the single target positioning problem, the sensor formation geometry that maximizes the FIM determinant.

The D-optimality criterion for the design of optimal sensor configurations is commonly used in the literature. Other indicators, like the A or E-optimality criteria, are also used by many authors. The D-optimality criterion minimizes the volume of the uncertainty ellipsoid for the target estimate, whereas the A-optimality criterion, which consists in minimizing the trace of the CRLB matrix, suppresses the average variance of the estimate, and the E-optimality design, which consists in minimizing the largest eigenvalue of the CRLB matrix, minimizes the length of the largest axis of the same ellipsoid [35]. It is interesting to notice that these other indicators are also optimized with the methodology adopted in this paper, as can be deduced from the Equation (21) of the optimal FIM.

An important advantage of using the D-optimality criterion is that it is invariant under scale changes in the parameters and linear transformations of the output, whereas A-optimality and E-optimality are affected by these transformations. However, if the global optimal is not obtained, the D-optimality criterion may yield some errors, because the information in one dimension can be improved rapidly, while we may have no practical information in the other. This problem can be avoided with the A/E-optimality criteria, [44]. Despite the above, for simplicity reasons, the D-optimality criterion is used, because the A/E-optimality criteria require the computation of the inverse of the FIM. Furthermore, the global optimal solutions defined in this work not only maximize the D-optimality criterion, for they provide the optimal FIM and the maximum possible determinant for each of the targets, but they also minimize the A/E-optimality criteria, while avoiding tedious computations.

### 3.2. Optimal Sensor Configurations

The optimal formations can be obtained analytically from the system defined in Equation (23). For the problem at hand, Equation (23) can be rewritten as follows:

(24) $$\begin{array}{l} \sum_{i=1}^{n}\frac{p_{ix}^{2}}{r_{i}^{2}}=\sum_{i=1}^{n}\cos^{2}\left(\alpha_{i}\right)=\frac{n}{2}\\ \sum_{i=1}^{n}\frac{p_{iy}^{2}}{r_{i}^{2}}=\sum_{i=1}^{n}\sin^{2}\left(\alpha_{i}\right)=\frac{n}{2}\\ \sum_{i=1}^{n}\frac{p_{ix}p_{iy}}{r_{i}^{2}}=\sum_{i=1}^{n}\sin\left(\alpha_{i}\right)\cos\left(\alpha_{i}\right)=0 \end{array}$$

Using by now classical terminology, the sensor formation must be first and second moment balanced. Then, from Equation (24), all the necessary conditions to determine an optimal formation are defined. One simple and intuitive configuration arises by noticing the orthogonality relations for sines and cosines from Fourier analysis [45].

(25) $$\begin{array}{c} \sum_{i=0}^{n-1}\cos^{2}\left(\frac{2\pi }{n}\cdot i\right)=\frac{n}{2}\\ \sum_{i=0}^{n-1}\sin^{2}\left(\frac{2\pi }{n}\cdot i\right)=\frac{n}{2}\\ \sum_{n=0}^{n-1}\cos\left(\frac{2\pi }{n}\cdot i\right)\sin\left(\frac{2\pi }{n}\cdot i\right)=0\\ \sum_{i=0}^{n-1}\cos\left(\frac{2\pi }{n}\cdot i\right)=0\\ \sum_{i=0}^{n-1}\sin\left(\frac{2\pi }{n}\cdot i\right)=0 \end{array}$$

Thus, the maximum FIM determinant is obtained with the sensor network regularly distributed around the target position. Obviously, an infinite number of solutions may be obtained by rotating the sensors rigidly around the target, that is, by allowing the above angles to become *2πi*/*n* + *α_c_*; *i* = 0, 1, …, *n* − 1, where *α_c_* is a fixed, but arbitrary, angle in [0, 2*π*]. This fact shows clearly that the optimal solution depends directly on the angles that the range vectors form among them.

It is important to remark on one important feature of the optimal solutions that can be computed based on the analysis explained above. If two disjoint sets of *n* and *m* sensors each are optimally placed, the resulting formation of *n* + *m* sensors is also optimal. Therefore, new higher order optimal solutions can be obtained by combining reduced order optimal configurations.

#### 3.2.1. Examples of Optimal Sensor Placement

In what follows, two examples are shown to illustrate the methodology developed for optimal sensor placement for single target positioning. For both examples, the sensors are considered to be placed at a distance of 20 m with respect to the target.

Clearly, in order for the information about the optimal configurations to be useful, one must check if the logarithm of the determinant of the FIM meets desired specifications. To this effect, and for comparison purposes, the determinant of the FIM obtained for a number of hypothetical target points (based on a fixed optimal sensor configuration corresponding to a well-defined scenario) will, at times, be computed by allowing these points to be on a grid in a finite spatial region, 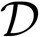. This will allow one to evaluate how good the sensor formation is in terms of yielding accurate localization of the real target, in comparison with the performance localization accuracy that is possible for any hypothetical target (different from the real one) positioned anywhere in 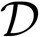. For the sake of clarity, and with an obvious abuse of notation, that determinant will be denoted, viewed as a function of its argument in 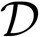, simply as 
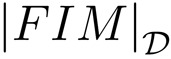
. In this work, 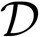 will always be a rectangle in ℜ^2^. The same comments apply to 
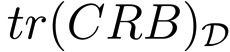
 for the trace of the CRB matrix in 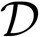.

**Figure 2.**
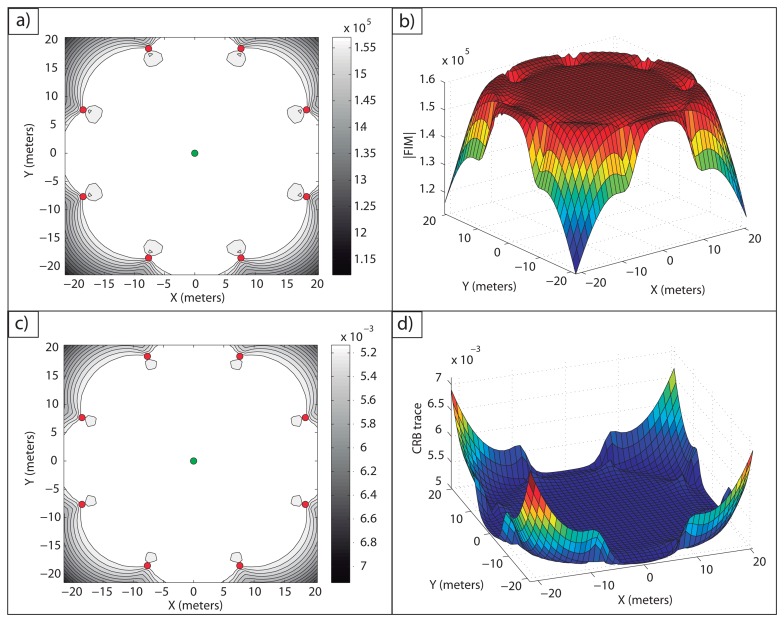
Optimal sensor placement for eight sensors. In (**a**), the level curves of 
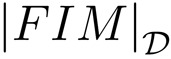
 are shown (lighter regions, larger accuracy); and in (**b**), its magnitude in 3D for 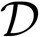. In (**c**), the level curves of 
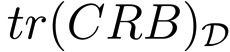
 are shown (lighter regions, larger accuracy); and in (**d**) its magnitude in 3D for 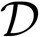.

**Example 1**: In Figure 2a, an optimal sensor formation of eight sensors regularly distributed around the target is shown, with *σ* = 0.1 *m*. It can be noticed how the maximum FIM determinant is obtained at the target position (lighter regions, larger accuracy), taking the theoretical maximum value, *n*^2^/(*σ*^4^·4) = 16·10^4^*m*^−4^. In Figure 2b, the magnitude of 
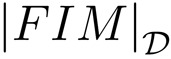
 in 3D for 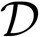 is shown.

In Figure 2c,d, the level curves of 
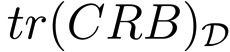
 (lighter regions, larger accuracy) and the representation of its magnitude in 3D for 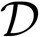 are shown, respectively. This shows the equivalence between the maximum determinant and the minimum CRB trace for the optimal sensor placement problem considered. The minimum trace of the CRB is obtained at the target position, taking its theoretical minimum value as well, *tr*(*CRB*) = *σ*^2^·4/*n* = 0.05 *m*^2^. This correspondence between the minimum trace of the CRB (A-optimum design) and the maximum FIM determinant (D-optimum design) is clear from the fact that the optimal FIM is a diagonal matrix with all the eigenvalues being equal.

**Figure 3.**
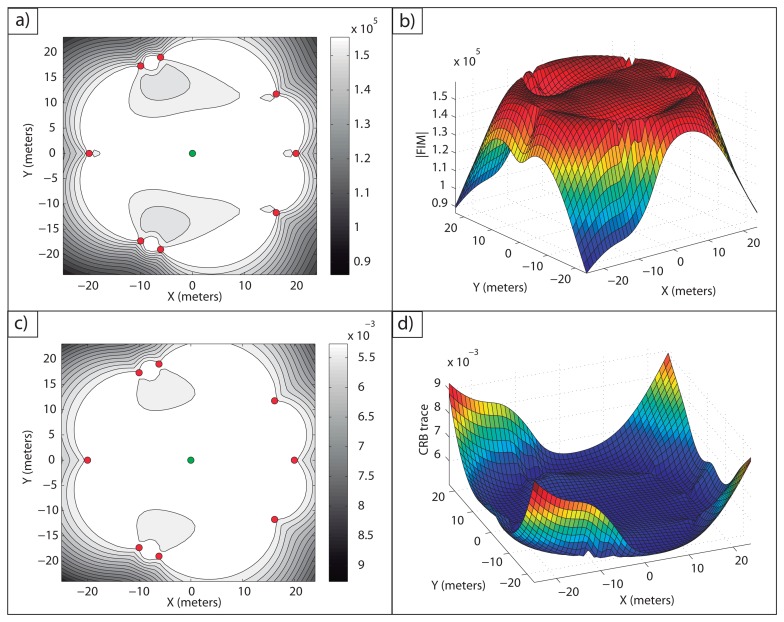
Optimal sensor configuration for eight sensors formed by the combination of a five sensor regular formation and a three sensor regular formation. In (**a**), the level curves of 
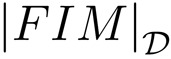
 are shown and, in (**b**), its magnitude in 3D for 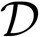. In (**c**), the level curves of 
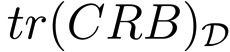
 are shown and, in (d), its magnitude in 3D for 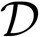.

**Example 2:** In this example, one important feature of the optimal solutions that can be computed based on the analysis explained above is highlighted. As aforementioned, if two disjoint sets of *n* and *m* sensors each are optimally placed, the resulting formation of *n* + *m* sensors is also optimal. Therefore, new higher order optimal solutions can be obtained by combining reduced order optimal configurations. This is a consequence of considering the measurements to be independent. It can be seen in Figure 3 how the combination of a five sensor regular formation with a three sensor regular formation provides another optimal formation in which the theoretical maximum accuracy for eight sensors is obtained at the target location, that is, |*FIM*| = 16·10^4^*m*^−4^.

Notice how for both optimal formations of Examples 1 and 2, the maximum theoretical accuracy is obtained at the target position, and thus, they are equivalent. However, the determinant, 
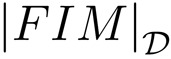
, over the region 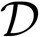, shows different accuracies in some parts of the area studied. This fact will be of great importance to determine the most adequate optimal formation when the target position is known with uncertainty or when the average accuracy over a given area must be maximized.

## 4. Problem Formulation for Multiple Target Positioning

To address the problem of multiple target positioning we start by introducing the notation *q_k_* = [*q_kx_*, *q_ky_*]*^T^*; *k* = 1, 2,.., *m* which denotes the position of the *k* − *th* target to be positioned in {*I*}. Further, denote by *p_i_* = [*p_ix_*, *p_iy_*]*^T^*; *i* = 1, 2,.., *n* the position of the *i* − *th* ranging sensor, also in {*I*}. Let *r_ki_*(*q*) = |*q_k_* − *p_i_*| (abbreviated *r_ki_*) be the distance (range) between the target, *q_k_*, and the *i* – *th* sensor, where | · | denotes the Euclidean norm, and *ω_ik_* is the corresponding measurement noise, as explained in Section 2. With this notation, the FIM for the *k* − *th* target becomes:

(26) $$FIM_{k}=\frac{1}{\sigma^{2}}\sum_{i=1}^{n}\left(\begin{array}{cc} {\left(\frac{\partial \left|q_{k}-p_{i}\right|}{\partial q_{x}}\right)}^{2} & \left(\frac{\partial \left|q_{k}-p_{i}\right|}{\partial q_{y}}\right)\;\left(\frac{\partial \left|q_{k}-p_{i}\right|}{\partial q_{x}}\right)\\ \left(\frac{\partial \left|q_{k}-p_{i}\right|}{\partial q_{x}}\right)\;\left(\frac{\partial \left|q_{k}-p_{i}\right|}{\partial q_{y}}\right) & {\left(\frac{\partial \left|q_{k}-p_{i}\right|}{\partial q_{y}}\right)}^{2} \end{array}\right)$$

where *k* ∈ {1, …, *m*}, with *m* being the number of targets.

As explained before, the determinant of the FIM corresponding to a single target is an indicator of the performance in positioning that can be achieved with a given sensor configuration. To tackle the multiple target problem we adopt, in this paper, an indicator that is the sum of the logarithms of the determinants of the FIMs of each target. Accordingly, the multiple target positioning problem is formulated as that of computing:

(27) $${\bar{p}}^{*}=\arg\underset{\bar{p}}{\max}\sum_{k=1}^{m}\log\left|FIM_{k}\right|$$

where *m* is the number of targets involved in the multiple target positioning task and *p̅* is the vector of sensor positions.

At this point it is important to notice that in a number of situations, maximizing this indicator yields sensor formations with the following important property: the determinant of the FIM of each and every target is equal to the maximum value obtained by considering each target in isolation (that is, by solving a single target positioning problem for each of the targets). Stated differently, there exist sensor formations that can be computed efficiently and yield optimal performance for all targets simultaneously. Example 3 below will show how this can be proven analytically for the case of 2 targets and 4 sensors (the same result applies to other relatively simple combinations of targets and sensors). For more complex target/sensor arrangements the analytical proof yields conditions for the sensor configuration that may be extremely hard to interpret geometrically. In these cases, the maximization of the indicator adopted can be done using numerical optimization tools that borrow from convex optimization theory.

It is necessary to point out that although the terms, *convexity* and *convex optimization tools*, are often used along this work, actually, the concavity of the log determinant of the FIM is searched, since a maximization problem is studied. The cost criterion used involves terms with the logarithm of the determinant of a Fisher Information Matrix, the latter “living naturally” in the set of positive definite matrices. It is well known that log |*F*|, where F is a positive definite matrix, is a “well behaved” function, that is concave in the set of positive definite matrices; see, for example, [34], Chapter 3. However, it is not necessarily true that log |*F*| is concave in the parameter search space (in the present case, the parameters that define the geometric configuration of the sensor network). Thus, each case must be examined carefully. In this sense, the simplest problem of a two sensor network is studied, because it is possible to demonstrate analytically and easily the concavity of the optimality criterion and, thus, that a global unique solution may be obtained with numerical search methods; see, also, the work in [35] on the D-optimality criterion.

In principle, to uniquely compute the position of a target in 2D, three different ranges from noncollinear sensors must be obtained. Notice, however, that the interest of the present study is not in deriving position estimators, but rather, in understanding what is the best performance (in terms of target positioning) that can be achieved with any unbiased estimator; that is, the objective is to understand how the target position impacts on the optimal sensor geometry. Notice now, from a practical standpoint, that if extra information about the target is available, then one can actually compute its position by using a smaller number of sensors. This can be easily understood in the case of a target in 3D using three sensors located in a plane. In this situation, knowledge that the target is above or below the plane is sufficient to find its position using three sensors and not four, as would be necessary if that information was not available. Identical comments apply to positioning in 2D using only two sensors, if extra information will allow us to disambiguate between the two possible solutions. The above reasoning justifies the study of the limits of performance of any estimator that relies on two sensors and, at the same time, incorporates extra information, allowing it to disambiguate between two possible solutions. Because the solutions exhibit symmetry (with respect to the array of sensors), there is no difference in the accuracy (as evaluated by the covariance of the estimation error) with which each of them can be computed. This justifies the demonstration example included.

For this purpose, the FIM for two sensors is defined:

(28) $$\text{FIM}=\frac{1}{\sigma^{2}}\left(\begin{array}{cc} \cos^{2}\left(\alpha_{1}\right)+\cos^{2}\left(\alpha_{2}\right) & \cos\left(\alpha_{1}\right)\sin\left(\alpha_{1}\right)+\cos\left(\alpha_{2}\right)\sin\left(\alpha_{2}\right)\\ \cos\left(\alpha_{1}\right)\sin\left(\alpha_{1}\right)+\cos\left(\alpha_{2}\right)\sin\left(\alpha_{2}\right) & \sin^{2}\left(\alpha_{1}\right)+\sin^{2}\left(\alpha_{2}\right) \end{array}\right)$$

Straightforward computations show that the determinant of Equation (28) becomes:

(29) $$\left|\text{FIM}\right|=\frac{1}{\sigma^{4}}\sin^{2}\left(\alpha_{12}\right)$$

where *α*_12_ = *α*_2_ − *α*_1_. The optimal sensor configuration will be defined as the one which maximizes the logarithm of Equation (29), which depends only on the angle that the range vectors form between them.

It is important to remark that the concavity of the logarithm of the FIM determinant is restricted to positive definite matrices [34]; therefore, the domain of the logarithm of Equation (29) cannot contain FIM determinants equal to zero, *i.e.*, the sensors and target cannot be collinear and, thus, *α*_12_ ∈ ]0, *π*[. For the domain, *α*_12_ ∈ ]*π*, 2*π*[, the solutions are equivalent and define the same formations only by rotating them the adequate angle. The first derivative of the logarithm of Equation (29) with respect to the angle, *α*_12_, is computed, which yields:

(30) $$\frac{\partial \log\left|\text{FIM}\right|}{\partial \alpha_{12}}=\frac{2\sin(\alpha_{12})\cos(\alpha_{12})}{\sigma^{4}\cdot \sin^{2}(\alpha_{12})}$$

The second derivative yields:

(31) $$\frac{\partial^{2}\log\left|\text{FIM}\right|}{\partial \alpha_{12}^{2}}=\frac{-2\sin^{4}(\alpha_{12})-2\sin^{2}(\alpha_{12})\cos^{2}(\alpha_{12})}{\sigma^{4}\cdot \sin^{4}(\alpha_{12})}=\frac{-2}{\sigma^{4}\cdot \sin^{2}(\alpha_{12})}$$

which is negative for the whole domain. Therefore, Equation (29) is a concave function; so, convex optimization tools can be employed to determine the optimal sensor configurations. In the analysis for a larger number of sensors, the initial guess should be chosen with care to avoid possible local maxima, due to the fact that the concavity of the criterion for an arbitrary number of sensors has not been demonstrated analytically. Although concavity of the criterion used is quite convenient from a computational point of view, it is not essential to the methodology proposed in the paper, for the optimal sensor configurations can be obtained with the gradient optimization method described. The latter will be presented in Section 6, where the methodology adopted provides more than satisfactory results. Moreover, the multiple examples carried out for different initial guesses show the repeatability of the optimal solutions proposed.

For the sake of clarity, in the notation and demonstration of the forthcoming analysis, index *s* is used instead of index *i* in the summations. Thus, Equation (26) is rewritten in the following form:

(32) $$FIM_{k}=\frac{1}{\sigma^{2}}\sum_{s=1}^{n}\left(\begin{array}{c} \cos^{2}(\alpha_{sk})\\ \cos(\alpha_{sk})\sin(\alpha_{sk}) \end{array}\begin{array}{c} \cos\left(\alpha_{sk}\right)\sin(\alpha_{sk})\\ \sin^{2}(\alpha_{sk}) \end{array}\right)$$

for *s* ∈ {1, …, *n*} and *k* ∈ {1, …, *m*}.

To shed light into the multiple target positioning problem, the simplest case of two targets and an arbitrary number of sensors (but at least three sensors) is studied first. Then, the methodology is extended to the general problem of an arbitrary number of targets.

## 5. Two Target Positioning

The optimal solutions can be searched with convex optimization techniques; however, there exist several situations in which it is possible to define analytical solutions. In this section, the simplest of these situations, the two target positioning problem, is tackled. In this particular case, Equation (27) becomes:

(33) $${\bar{p}}^{*}=\arg\underset{\bar{p}}{\max}(\log\left|FIM_{1}\right|+\log\left|FIM_{2}\right|)$$

The summation of logarithms in Equation (33) is equivalent to:

(34) $$\log\left|FIM_{1}\right|+\log\left|FIM_{2}\right|=\log(\left|FIM_{1}\right|\cdot \left|FIM_{2}\right|)=\log(\left|FIM_{T}\right|)$$

where the meaning of |*FIM_T_*| is clear from the context. Moreover, the determinant of Equation (32) may be written as:

(35) $$\left|FIM_{k}\right|=\frac{1}{\sigma^{4}}\left(\sum_{s=1}^{n}\cos^{2}(\alpha_{sk}),-\sum_{s=1}^{n}\cos(\alpha_{sk})\sin(\alpha_{sk})\right)\cdot \left(\begin{array}{c} \sum_{s=1}^{n}\sin^{2}(\alpha_{sk})\\ \sum_{s=1}^{n}\cos(\alpha_{sk})\sin(\alpha_{sk}) \end{array}\right)$$

Thus, from Equations (34) and (35), Equation (33) yields:

(36) $${\bar{p}}^{*}=\arg\underset{\bar{p}}{\max}\log\frac{1}{\sigma^{4m}}\prod_{k=1}^{m}\left(\left(\sum_{s=1}^{n}\cos^{2}\left(\alpha_{sk}\right),-\sum_{s=1}^{n}\cos\left(\alpha_{sk}\right)\sin\left(\alpha_{sk}\right)\right)\cdot \left(\begin{array}{c} \sum_{s=1}^{n}\sin^{2}\left(\alpha_{sk}\right)\\ \sum_{s=1}^{n}\cos\left(\alpha_{sk}\right)\sin\left(\alpha_{sk}\right) \end{array}\right)\right)$$

where *k* = 1, …,*m*, and *m* is the number of targets involved in the task, in this particular case, *m* = 2. Equation (36) can be written in the following compact form:

(37) $$\log\left|FIM_{T}\right|=\log\left(\frac{1}{\sigma^{4m}}\prod_{k=1}^{m}{\vec{P}}_{k}\cdot {\vec{Q}}_{k}\right)$$

with:

$${\begin{array}{c} {\vec{P}}_{k}=\left(\begin{array}{c} \sum_{s=1}^{n}\cos^{2}(\alpha_{sk})\\ -\sum_{s=1}^{n}\cos(\alpha_{sk})\sin(\alpha_{sk}) \end{array}\right)\\ {\vec{Q}}_{k}=\left(\begin{array}{c} \sum_{s=1}^{n}\sin^{2}(\alpha_{sk})\\ \sum_{s=1}^{n}\cos(\alpha_{sk})\sin(\alpha_{sk}) \end{array}\right) \end{array}}^{T}$$

The optimal solution is defined by computing the derivatives of Equation (37) with respect to each sensor position coordinate, *p_ix_* and *p_iy_*, with *i* = 1, ⋯, *n*. The derivative of a dot product is defined by *∂*(*P⃗* · *Q⃗*) = *∂P⃗* · *Q⃗* + *P⃗* · *∂Q⃗*, and the derivative of a vector is defined by the derivatives of each of its elements, [*∂P⃗*_1_ ⋯ *∂P⃗_n_*]; so, the derivatives of each vector element with respect to each sensor position coordinate must be computed, too.

From the above, the derivative of Equation (37) with respect to the {*x_I_*} coordinate of sensor *i* yields:

$$\begin{array}{ll} \frac{\partial \log\left|FIM_{T}\right|}{\partial p_{ix}} & ={\left(\begin{array}{c} -\frac{2\cos\left(\alpha_{i1}\right)\sin^{2}\left(\alpha_{i1}\right)}{r_{i1}}\\ \frac{\sin^{3}\left(\alpha_{i1}\right)}{r_{i1}}-\frac{\cos^{2}\left(\alpha_{i1}\right)\sin\left(\alpha_{i1}\right)}{r_{i1}} \end{array}\right)}^{T}\left(\begin{array}{c} \sum_{s=1}^{n}\sin^{2}\left(\alpha_{s1}\right)\\ \sum_{s=1}^{n}\cos\left(\alpha_{s1}\right)\sin\left(\alpha_{s1}\right) \end{array}\right){\left|FIM_{1}\right|}^{-1}+\\ & {\left(\begin{array}{c} \sum_{s=1}^{n}\cos^{2}\left(\alpha_{s1}\right)\\ -\sum_{s=1}^{n}\cos\left(\alpha_{s1}\right)\sin\left(\alpha_{s1}\right) \end{array}\right)}^{T}\left(\begin{array}{c} \frac{2\cos\left(\alpha_{i1}\right)\sin^{2}\left(\alpha_{i1}\right)}{r_{i1}}\\ -\frac{\sin^{3}\left(\alpha_{i1}\right)}{r_{i1}}+\frac{\cos^{2}\left(\alpha_{i1}\right)\sin\left(\alpha_{i1}\right)}{r_{i1}} \end{array}\right){\left|FIM_{1}\right|}^{-1}+\\ & {\left(\begin{array}{c} -\frac{2\cos\left(\alpha_{i2}\right)\sin^{2}\left(\alpha_{i2}\right)}{r_{i2}}\\ \frac{\sin^{3}\left(\alpha_{i2}\right)}{r_{i2}}-\frac{\cos^{2}\left(\alpha_{i2}\right)\sin\left(\alpha_{i2}\right)}{r_{i2}} \end{array}\right)}^{T}\left(\begin{array}{c} \sum_{s=1}^{n}\sin^{2}\left(\alpha_{s2}\right)\\ \sum_{s=1}^{n}\cos\left(\alpha_{s2}\right)\sin\left(\alpha_{s2}\right) \end{array}\right){\left|FIM_{2}\right|}^{-1}+\\ & {\left(\begin{array}{c} \sum_{s=1}^{n}\cos^{2}\left(\alpha_{s2}\right)\\ -\sum_{s=1}^{n}\cos\left(\alpha_{s2}\right)\sin\left(\alpha_{s2}\right) \end{array}\right)}^{T}\left(\begin{array}{c} \frac{2\cos\left(\alpha_{i2}\right)\sin^{2}\left(\alpha_{i2}\right)}{r_{i2}}\\ -\frac{\sin^{3}\left(\alpha_{i2}\right)}{r_{i2}}+\frac{\cos^{2}\left(\alpha_{i2}\right)\sin\left(\alpha_{i2}\right)}{r_{i2}} \end{array}\right){\left|FIM_{2}\right|}^{-1} \end{array}$$

Similarly, the derivative of Equation (37) with respect to the {*y_I_*} coordinate of sensor *i* yields:

$$\begin{array}{ll} \frac{\partial \log\left|FIM_{T}\right|}{\partial p_{iy}} & ={\left(\begin{array}{c} \frac{2\cos^{2}\left(\alpha_{i1}\right)\sin\left(\alpha_{i1}\right)}{r_{i1}}\\ -\frac{\cos\left(\alpha_{i1}\right)\sin^{2}\left(\alpha_{i1}\right)}{r_{i1}}+\frac{\cos^{3}\left(\alpha_{i1}\right)}{r_{i1}} \end{array}\right)}^{T}\left(\begin{array}{c} \sum_{s=1}^{n}\sin^{2}\left(\alpha_{s1}\right)\\ \sum_{s=1}^{n}\cos\left(\alpha_{s1}\right)\sin\left(\alpha_{s1}\right) \end{array}\right){\left|FIM_{1}\right|}^{-1}+\\ & +{\left(\begin{array}{c} \sum_{s=1}^{n}\cos^{2}\left(\alpha_{s1}\right)\\ -\sum_{s=1}^{n}\cos\left(\alpha_{s1}\right)\sin\left(\alpha_{s1}\right) \end{array}\right)}^{T}\left(\begin{array}{c} -\frac{2\cos^{2}\left(\alpha_{i1}\right)\sin\left(\alpha_{i1}\right)}{r_{i1}}\\ +\frac{\cos\left(\alpha_{i1}\right)\sin^{2}\left(\alpha_{i1}\right)}{r_{i1}}-\frac{\cos^{3}\left(\alpha_{i1}\right)}{r_{i1}} \end{array}\right){\left|FIM_{1}\right|}^{-1}+\\ & +{\left(\begin{array}{c} \frac{2\cos^{2}\left(\alpha_{i2}\right)\sin\left(\alpha_{i2}\right)}{r_{i2}}\\ -\frac{\cos\left(\alpha_{i2}\right)\sin^{2}\left(\alpha_{i2}\right)}{r_{i2}}+\frac{\cos^{3}\left(\alpha_{i2}\right)}{r_{i2}} \end{array}\right)}^{T}\left(\begin{array}{c} \sum_{s=1}^{n}\sin^{2}\left(\alpha_{s2}\right)\\ \sum_{s=1}^{n}\cos\left(\alpha_{s2}\right)\sin\left(\alpha_{s2}\right) \end{array}\right){\left|FIM_{2}\right|}^{-1}+\\ & {\left(\begin{array}{c} \sum_{s=1}^{n}\cos^{2}\left(\alpha_{s2}\right)\\ -\sum_{s=1}^{n}\cos\left(\alpha_{s2}\right)\sin\left(\alpha_{s2}\right) \end{array}\right)}^{T}\left(\begin{array}{c} -\frac{2\cos^{2}\left(\alpha_{i2}\right)\sin\left(\alpha_{i2}\right)}{r_{i2}}\\ \frac{\cos\left(\alpha_{i2}\right)\sin^{2}\left(\alpha_{i2}\right)}{r_{i2}}-\frac{\cos^{3}\left(\alpha_{i2}\right)}{r_{i2}} \end{array}\right){\left|FIM_{2}\right|}^{-1} \end{array}$$

Straightforward computations allow one to rewrite the above derivatives as:

(38) $$\begin{array}{ll} \frac{\partial \log\left|FIM_{T}\right|}{\partial p_{ix}} & =-\frac{2\cos\left(\alpha_{i1}\right)\sin^{2}\left(\alpha_{i1}\right)}{r_{i1}}\sum_{s=1}^{n}\left(\sin^{2}\left(\alpha_{s1}\right)-\cos^{2}\left(\alpha_{s1}\right)\right){\left|FIM_{1}\right|}^{-1}+\\ & 2\left(\frac{\sin^{3}\left(\alpha_{i1}\right)}{r_{i1}}-\frac{\cos^{2}\left(\alpha_{i1}\right)\sin\left(\alpha_{i1}\right)}{r_{i1}}\right)\sum_{s=1}^{n}\cos\left(\alpha_{s1}\right)\sin\left(\alpha_{s1}\right){\left|FIM_{1}\right|}^{-1}-\\ & \frac{2\cos\left(\alpha_{i2}\right)\sin^{2}\left(\alpha_{i2}\right)}{r_{i2}}\sum_{s=1}^{n}\left(\sin^{2}\left(\alpha_{s2}\right)-\cos^{2}\left(\alpha_{s2}\right)\right){\left|FIM_{2}\right|}^{-1}+\\ & 2\left(\frac{\sin^{3}\left(\alpha_{i2}\right)}{r_{i2}}-\frac{\cos^{2}\left(\alpha_{i2}\right)\sin\left(\alpha_{i2}\right)}{r_{i2}}\right)\sum_{s=1}^{n}\cos\left(\alpha_{s2}\right)\sin\left(\alpha_{s2}\right){\left|FIM_{2}\right|}^{-1} \end{array}$$

(39) $$\begin{array}{ll} \frac{\partial \log\left|FIM_{T}\right|}{\partial p_{iy}} & =\frac{2\cos\left(\alpha_{i1}\right)\sin^{2}\left(\alpha_{i1}\right)}{r_{i1}}\sum_{s=1}^{n}\left(\sin^{2}\left(\alpha_{s1}\right)-\cos^{2}\left(\alpha_{s1}\right)\right){\left|FIM_{1}\right|}^{-1}+\\ & 2\left(\frac{\cos^{3}\left(\alpha_{i1}\right)}{r_{i1}}-\frac{\cos\left(\alpha_{i1}\right)\sin^{2}\left(\alpha_{i1}\right)}{r_{i1}}\right)\sum_{s=1}^{n}\cos\left(\alpha_{s1}\right)\sin\left(\alpha_{s1}\right){\left|FIM_{1}\right|}^{-1}+\\ & 2\frac{\cos\left(\alpha_{i2}\right)\sin^{2}\left(\alpha_{i2}\right)}{r_{i2}}\sum_{s=1}^{n}\left(\sin^{2}\left(\alpha_{s2}\right)-\cos^{2}\left(\alpha_{s2}\right)\right){\left|FIM_{2}\right|}^{-1}+\\ & 2\left(\frac{\cos^{3}\left(\alpha_{i2}\right)}{r_{i2}}-\frac{\cos\left(\alpha_{i2}\right)\sin^{2}\left(\alpha_{i2}\right)}{r_{i2}}\right)\sum_{s=1}^{n}\cos\left(\alpha_{s2}\right)\sin\left(\alpha_{s2}\right){\left|FIM_{2}\right|}^{-1} \end{array}$$

The optimal sensor configuration may be defined by making these equations equal to zero. Equations (38) and (39) can be rewritten again as:

(40) $$\begin{array}{ll} \frac{\partial \log\left|FIM_{T}\right|}{\partial p_{ix}} & =\frac{\sin\left(\alpha_{i1}\right)}{r_{i1}}\left[\sin\left(2\alpha_{i1}\right)\sum_{s=1}^{n}\cos\left(2\alpha_{s1}\right)-\cos\left(2\alpha_{i1}\right)\sum_{s=1}^{n}\sin\left(2\alpha_{s1}\right)\right]{\left|FIM_{1}\right|}^{-1}+\\ & \frac{\sin\left(\alpha_{i2}\right)}{r_{i2}}\left[\sin\left(2\alpha_{i2}\right)\sum_{s=1}^{n}\cos\left(2\alpha_{s2}\right)-\cos\left(2\alpha_{i2}\right)\sum_{s=1}^{n}\sin\left(2\alpha_{s2}\right)\right]{\left|FIM_{2}\right|}^{-1}=0 \end{array}$$

(41) $$\begin{array}{ll} \frac{\partial \log\left|FIM_{T}\right|}{\partial p_{iy}} & =\frac{\cos\left(\alpha_{i1}\right)}{r_{i1}}\left[-\sin\left(2\alpha_{i1}\right)\sum_{s=1}^{n}\cos\left(2\alpha_{s1}\right)+\cos\left(2\alpha_{i1}\right)\sum_{s=1}^{n}\sin\left(2\alpha_{s1}\right)\right]{\left|FIM_{1}\right|}^{-1}+\\ & \frac{\cos\left(\alpha_{i2}\right)}{r_{i2}}\left[-\sin\left(2\alpha_{i2}\right)\sum_{s=1}^{n}\cos\left(2\alpha_{s2}\right)+\cos\left(2\alpha_{i2}\right)\sum_{s=1}^{n}\sin\left(2\alpha_{s2}\right)\right]{\left|FIM_{2}\right|}^{-1}=0 \end{array}$$

Now, Equations (40) and (41) can be seen as dot products:

(42) $$\frac{\partial \log\left|FIM_{T}\right|}{\partial p_{ix}}=\left(\begin{array}{cc} \frac{\sin(\alpha_{i1})}{r_{i1}}\cdot v_{i1} & \frac{\sin(\alpha_{i2})}{r_{i2}}\cdot v_{i2} \end{array}\right)\cdot \left(\begin{array}{c} \frac{1}{\left|FIM_{1}\right|}\\ \frac{1}{\left|FIM_{2}\right|} \end{array}\right)=0$$

(43) $$\frac{\partial \log\left|FIM_{T}\right|}{\partial p_{iy}}=\left(\begin{array}{cc} -\frac{\cos(\alpha_{i1})}{r_{i1}}\cdot v_{i1} & -\frac{\cos(\alpha_{i2})}{r_{i2}}\cdot v_{i2} \end{array}\right)\cdot \left(\begin{array}{c} \frac{1}{\left|FIM_{1}\right|}\\ \frac{1}{\left|FIM_{2}\right|} \end{array}\right)=0$$

where $v_{ik}=\sin\left(2\alpha_{ik}\right)\sum_{s=1}^{n}\cos\left(2\alpha_{sk}\right)-\cos\left(2\alpha_{ik}\right)\sum_{s=1}^{n}\sin\left(2\alpha_{sk}\right)$ for *k* =1, 2. It is easy to check that if the dot products are equal to zero, then the vectors are orthogonal. Therefore, vectors:

$$\begin{array}{c} V_{1}=\left(\begin{array}{cc} \frac{\sin(\alpha_{i1})}{r_{i1}}\cdot v_{i1} & \frac{\sin(\alpha_{i2})}{r_{i2}} \end{array}\cdot v_{i2}\right)\\ V_{2}=\left(\begin{array}{cc} -\frac{\cos(\alpha_{i1})}{r_{i1}}\cdot v_{i1} & -\frac{\cos(\alpha_{i2})}{r_{i2}}\cdot v_{i2} \end{array}\right) \end{array}$$

are equivalent, and then, *V*_1_(1)/*V*_2_(1) = *V*_1_(2)/*V*_2_(2), it is:

(44) $$\frac{-\sin(\alpha_{i1})\cdot r_{i1}\cdot v_{i1}}{\cos(\alpha_{i1})\cdot r_{i1}\cdot v_{i1}}=\frac{-\sin(\alpha_{i2})\cdot r_{i2}\cdot v_{i2}}{\cos(\alpha_{i2})\cdot r_{i2}\cdot v_{i2}}\to \tan(\alpha_{i1})=\tan(\alpha_{i2})$$

Equation (44) holds when *α_i_*_1_ = *α_i_*_2_ + *c* · *π*, with *c* being any natural number. This condition means that all the sensors must lie in the line joining the two targets, since Equation (44) must hold for all sensors; therefore, this solution can be discarded, because, obviously, it is not an optimal solution.

It is important to notice from Equations (42) and (43) that the vector, ${\left[\begin{array}{cc} \frac{1}{\left|FIM_{1}\right|}, & \frac{1}{\left|FIM_{2}\right|} \end{array}\right]}^{T}$, has its elements always different from zero and positive; so, the only possible solution is that vectors *V*_1_ and *V*_2_ are equal to zero. A closer look at these vectors shows that the only possible condition for them to be zero is that *v_ik_* is equal to zero.

(45) $$v_{ik}=\sin\left(2\alpha_{ik}\right)\sum_{s=1}^{n}\cos\left(2\alpha_{sk}\right)-\cos\left(2\alpha_{ik}\right)\sum_{s=1}^{n}\sin\left(2\alpha_{sk}\right)=0$$

With *k* = 1, 2. Now, Equation (45) can be seen again as a dot product between two vectors:

(46) $$\left(\begin{array}{cc} \sum_{s=1}^{n}\cos(2\alpha_{sk}) & \sum_{s=1}^{n}\sin(2\alpha_{sk}) \end{array}\right)\cdot \left(\begin{array}{cc} \sin(2\alpha_{ik}) & -\cos(2\alpha_{ik}) \end{array}\right)=0$$

This equation must hold for each sensor and each target. It is clear that if both vectors are different from zero, Equation (46) implies that the vectors are orthogonal. For a given optimal sensor configuration, the first array of the dot product of Equation (46) is constant for all sensors and a given target *k*. The second array of Equation (46) defines the orientation of the sensor, *i*, with respect to a target, *k*, and this orientation should be the same for all sensors with respect to target *k*, something that is not optimal for a single target and that is impossible to satisfy for more than one target. Hence, the solution of two orthogonal vectors is discarded, and the only valid solution is the one in which one of the vectors of Equation (46) is the null vector. A simple look at Equation (46) shows that:

$$\left(\begin{array}{cc} \cos(2\alpha_{ik}) & \sin(2\alpha_{ik}) \end{array}\right)\neq (\begin{array}{cc} 0 & 0 \end{array})$$

and therefore, the optimality condition is:

(47) $$\left(\begin{array}{cc} \sum_{s=1}^{n}\cos\left(2\alpha_{sk}\right) & \sum_{s=1}^{n}\sin\left(2\alpha_{sk}\right) \end{array}\right)=\left(\begin{array}{cc} 0 & 0 \end{array}\right)$$

Therefore, to obtain the maximum possible accuracy, the sensor network must be second moment balanced with respect to both targets:

(48) $$\begin{array}{l} \sum_{s=1}^{n}\cos\left(2\alpha_{sk}\right)=\sum_{s=1}^{n}\left(\cos^{2}\left(\alpha_{sk}\right)-\sin^{2}\left(\alpha_{sk}\right)\right)=0\\ \sum_{s=1}^{n}\sin\left(2\alpha_{sk}\right)=\sum_{s=1}^{n}2\cos\left(\alpha_{sk}\right)\sin\left(\alpha_{sk}\right)=0 \end{array}$$

Equation (47) is valid for any number of sensors. This solution defines optimal sensor formations that provide the theoretical maximum accuracy for both targets at the same time; so, it is not necessary to define a tradeoff solution, *i.e.*, the same optimality conditions defined for single target positioning can always hold for the two target problem. Moreover, it has been shown that this is the only valid solution for this particular case. For more than two targets, Equation (47) may not be the only valid solution, as shown in the next section. It is possible that the maximum theoretical accuracy for all the targets cannot be obtained. In this case, a tradeoff solution must be adopted.

### 5.1. Example of Optimal Sensor Placement for Two Target Positioning

**Example 3:** This example illustrates the case of a four sensor network for the positioning of two targets with no constraints in the sensor placement and with *σ* = 0.1 *m*. The targets are considered to be placed at *q*_1_ = [20, 0]*^T^m* and *q*_2_ = [−20, 0]*^T^m*.

**Figure 4.**
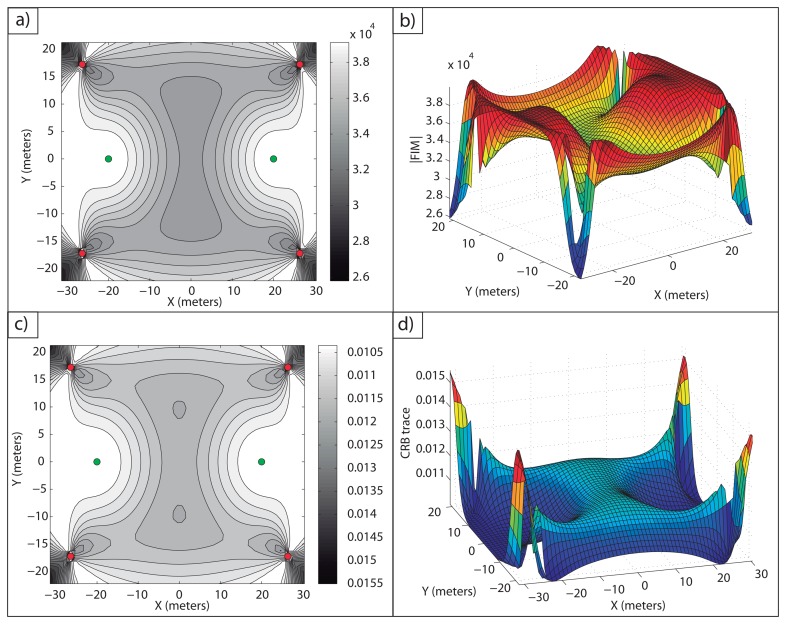
Optimal four sensor formation for two target positioning. In (**a**), the level curves of 
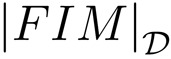
 in ℜ^2^ are shown; and in (**b**), its magnitude in 3D for 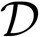. Similarly, in (**c**), 
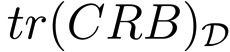
 is shown; and in (**d**), its magnitude in 3D for 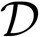.

The optimal sensor formation is defined by the positions listed in Table 1 and shown in Figure 4a, which provides the maximum possible FIM determinant, |*FIM*| = *n*^2^/(*σ*^4^ · 4) *m*^−4^, for each target. This optimal formation may also be computed with the gradient optimization algorithm described in Section 6. For the example at hand, the initial guess may be arbitrary, since the only feasible solution provides the theoretical maximum FIM determinant for both targets, as demonstrated above. In Figure 4c, notice how the formation provides also the minimum CRB trace, *tr*(*CRB*) = *σ*^2^·4/*nm*^2^, at the target positions.

In Figure 4b,d, the maximum values of the FIM determinant and the minimum values of the CRB trace, respectively, are over the target positions. Notice how the design condition in Equation (47) is achieved by the present formation for both targets. For *q*_1_ = [20, 0]*^T^m*, the condition in Equation (47) becomes:

$$\begin{array}{ll} \sum_{i=1}^{n}2\cos\left(\alpha_{i1}\right)\sin\left(\alpha_{i1}\right)= & \left(\frac{20+26.39}{49.48}\right)\left(\frac{17.22}{49.48}\right)+\left(\frac{20+26.39}{49.48}\right)\left(\frac{-17.22}{49.48}\right)\\ & \left(\frac{20-26.39}{18.37}\right)\left(\frac{17.22}{18.37}\right)+\left(\frac{20-26.39}{18.37}\right)\left(\frac{-17.22}{18.37}\right)=0 \end{array}$$

$$\begin{array}{ll} \sum_{i=1}^{n}\left(\cos^{2}(\alpha_{i1})-\sin^{2}(\alpha_{i1})\right)= & {\left(\frac{20+26.39}{49.48}\right)}^{2}+{\left(\frac{20+26.39}{49.48}\right)}^{2}+{\left(\frac{20-26.39}{18.37}\right)}^{2}\\ & +{\left(\frac{20-26.39}{18.37}\right)}^{2}-{\left(\frac{17.22}{49.48}\right)}^{2}-{\left(\frac{-17.22}{49.48}\right)}^{2}\\ & -{\left(\frac{17.22}{18.37}\right)}^{2}-{\left(\frac{-17.22}{18.37}\right)}^{2}=0 \end{array}$$

And for *q*_2_ = [−20, 0]*^T^m*:

$$\begin{array}{ll} \sum_{i=1}^{n}2\cos\left(\alpha_{i1}\right)\sin\left(\alpha_{i1}\right)= & \left(\frac{-20+26.39}{18.37}\right)\left(\frac{17.22}{18.37}\right)+\left(\frac{-20+21.21}{18.37}\right)\left(\frac{-17.22}{18.37}\right)\\ & \left(\frac{-20-26.39}{49.48}\right)\left(\frac{17.22}{49.48}\right)+\left(\frac{-20-26.39}{49.48}\right)\left(\frac{-17.22}{49.48}\right)=0 \end{array}$$

$$\begin{array}{ll} \sum_{i=1}^{n}\left(\cos^{2}\left(\alpha_{i1}\right)-\sin^{2}\left(\alpha_{i1}\right)\right)= & {\left(\frac{-20+26.39}{18.37}\right)}^{2}+{\left(\frac{-20+26.39}{18.37}\right)}^{2}+{\left(\frac{-20-26.39}{49.48}\right)}^{2}\\ & +{\left(\frac{-20-26.39}{49.48}\right)}^{2}-{\left(\frac{17.22}{18.37}\right)}^{2}-{\left(\frac{-17.22}{18.37}\right)}^{2}\\ & -{\left(\frac{17.22}{49.48}\right)}^{2}-{\left(\frac{-17.22}{49.48}\right)}^{2}=0 \end{array}$$

Therefore, it is easy to check that the optimal formation that provides the theoretical maximum FIM determinant, and also the theoretical minimum CRB trace, is obtained when Equation (47) holds for each of the targets. Moreover, the optimal formation is computed very fast; the computation time for this particular example was 0.0468 s (in a laptop with an Intel Centrino 2 with 4 Gb RAM and running MS Windows 7 Operative System), so the approach is suitable to be used in a practical scenario as the mission unfolds.

## 6. *m* Target Positioning

Once the analytical solution for the two target positioning problem has been defined, the analysis can be extended for an arbitrary number of targets, that is:

(49) $${\bar{p}}^{*}=\arg\underset{\bar{p}}{\max}\left(\log\left|FIM_{1}\right|+\cdots +\log\left|FIM_{m}\right|\right)=\text{arg}\underset{\bar{p}}{\max}\log\left|FIM_{T}\right|$$

From Equation (49), it is possible to obtain the gradient equations needed to define the optimal sensor configurations, in a manner similar to what was done to obtain Equations (40) and (41).

(50) $$\begin{array}{ll} \frac{\partial \log\left|FIM_{T}\right|}{\partial p_{ix}} & =\frac{\sin\left(\alpha_{i1}\right)}{r_{i1}\left|FIM_{1}\right|}\left[\sin\left(2\alpha_{i1}\right)\sum_{s=1}^{n}\cos\left(2\alpha_{i1}\right)-\cos\left(2\alpha_{i1}\right)\sum_{s=1}^{n}\sin\left(2\alpha_{i1}\right)\right]+\cdots \\ & +\frac{\sin\left(\alpha_{im}\right)}{r_{im}\left|FIM_{m}\right|}\left[\sin\left(2\alpha_{im}\right)\sum_{s=1}^{n}\cos\left(2\alpha_{sm}\right)-\cos\left(2\alpha_{im}\right)\sum_{s=1}^{n}\sin\left(2\alpha_{sm}\right)\right]=0 \end{array}$$

(51) $$\begin{array}{ll} \frac{\partial \log\left|FIM_{T}\right|}{\partial p_{iy}} & =\frac{\cos\left(\alpha_{i1}\right)}{r_{i1}\left|FIM_{1}\right|}\left[-\sin\left(2\alpha_{i1}\right)\sum_{s=1}^{n}\cos\left(2\alpha_{s1}\right)+\cos\left(2\alpha_{i1}\right)\sum_{s=1}^{n}\sin\left(2\alpha_{s1}\right)\right]+\cdots \\ & +\frac{\cos\left(\alpha_{im}\right)}{r_{im}\left|FIM_{m}\right|}\left[-\sin\left(2\alpha_{im}\right)\sum_{s=1}^{n}\cos\left(2\alpha_{sm}\right)+\cos\left(2\alpha_{im}\right)\sum_{s=1}^{n}\sin\left(2\alpha_{sm}\right)\right]=0 \end{array}$$

Again, Equations (50) and (51) can be written as dot products between two vectors:

(52) $$\frac{\partial \log\left|FIM_{T}\right|}{\partial p_{ix}}=\left(\begin{array}{ccc} \frac{\sin\left(\alpha_{i1}\right)}{r_{i1}}\cdot v_{i1} & \cdots & \frac{\sin\left(\alpha_{im}\right)}{r_{im}}.v_{im} \end{array}\right)\left(\begin{array}{c} \frac{1}{\left|FIM_{1}\right|}\\ \vdots \\ \frac{1}{\left|FIM_{m}\right|} \end{array}\right)=0$$

(53) $$\frac{\partial \log\left|FIM_{T}\right|}{\partial p_{iy}}=\left(\begin{array}{ccc} -\frac{\cos\left(\alpha_{i1}\right)}{r_{i1}}\cdot v_{i1} & \cdots & -\frac{\cos\left(\alpha_{im}\right)}{r_{im}}.v_{im} \end{array}\right)\left(\begin{array}{c} \frac{1}{\left|FIM_{1}\right|}\\ \vdots \\ \frac{1}{\left|FIM_{m}\right|} \end{array}\right)=0$$

with $v_{ik}=\sin\left(2\alpha_{ik}\right)\sum_{s=1}^{n}\cos\left(2\alpha_{ik}\right)-\cos\left(2\alpha_{ik}\right)\sum_{s=1}^{n}\sin\left(2\alpha_{sk}\right)$. Equations (52) and (53) must hold for all the sensors of the network. It must be noticed that the above system of equations may have multiple solutions. Nevertheless, the solution given by Equation (47) is one of the valid solutions for Equations (52) and (53), which also provides the theoretical maximum FIM determinant for each target. Thus, if it is possible to obtain a sensor configuration where Equation (47) holds, then the minimum covariance error for each target estimate is obtained. This optimal configuration will provide the maximum FIM determinant, *n*^2^/*σ*^4^2^2^, as defined in Section 3.

However, Equation (47) may or may not be a solution for a given multiple target localization problem, depending on the configuration of the targets and on the number of sensors involved in the task. If it is not possible to define a sensor configuration for which Equation (47) is satisfied for all the targets, then a tradeoff solution must be adopted. These solutions can be obtained by resorting to multiple objective optimization tools, and the tradeoffs can be mission–dependent.

The above discussion shows that there may be tradeoffs involved in the precision with which each of the targets can be localized; to study them, as mentioned above, techniques that borrow from estimation theory and convex optimization are used. For the latter, the reader is referred to [31–34]. Thus, the aim is to determine the sensor configuration that yields, if possible, the maximum accuracy with which the position of the different targets can be estimated.

### 6.1. Gradient Optimization Algorithm for Optimal Sensor Placement

Once the gradients defined by Equations (50) and (51) have been computed for each target, they are combined to update the sensor configuration, so as to yield an increase in the specified convex combination of the logarithms of the FIM determinants. This computation is recursive, and the process finalizes when the optimal position is found or when an appropriate stop criterion is met. Checking that this algorithm behaves well for single and two target positioning is easy, for analytical solutions to the optimal sensor positions are available in Sections 3 and 5, respectively. For the multiple target localization problem, the initial guess may be a regular distribution around the mass center of the target group, with all the targets inside the sensor formation; although, this initial guess could be different, depending on the mission constraints and requirements. The cost function is, again:

(54) $${\bar{p}}^{*}=\arg\underset{\bar{p}}{\max}\sum_{k=1}^{m}\log\left|FIM_{k}\right|=\arg\underset{\bar{p}}{\max}\log\left|FIM_{\tau }\right|$$

where *k* = 1, ⋯,*m*, with *m* being the number of targets. The Armijo rule is used for the sensor placement update phase, yielding the following iterative gradient optimization algorithm.

1. For each target, its FIM is computed for the current sensor formation at iteration *t*, from which 
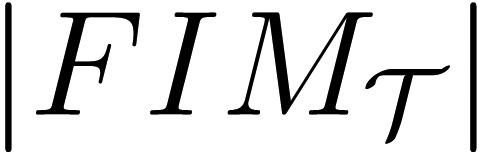
 is defined. The latter is the convex combination of the logarithms of the determinants, given by $\left|FIM_{\tau }\right|\left[t\right]=\sum_{k=1}^{m}\log\left|FIM_{k}\right|\left[t\right]$, where *m* is the number of targets.
2. Using Equations (50) and (51), the gradient of 
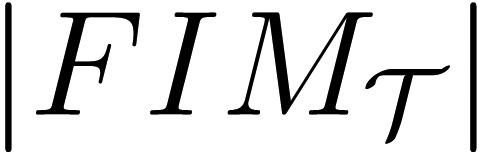
 [*t*] is computed, yielding ∇*_iξ_* 
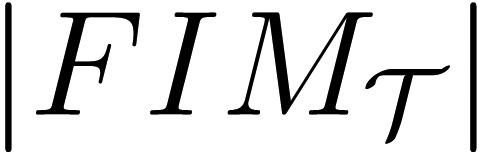
 [*t*] with *ξ* = *x*, *y*; and *i* = 1, …, *n*.
3. The sensor positions are updated according to the gradients: *p_iξ_* [*t*+ 1 ] = *p_iξ_* [*t*] + *μ^ζ^*^[^*^t^*^]^ ∇*_iξ_* 
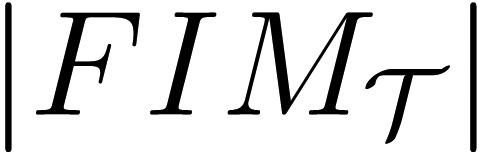
 [*t*], with *μ* ∈ 0, 1, *ζ* [0] = 1, and *ζ* [*t*] = *ζ* [*t* − 1] + 1.
4. If 
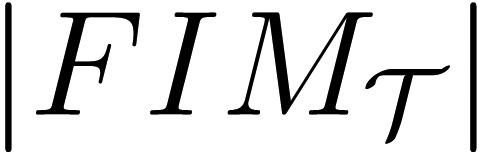
 [*t* −1] > 
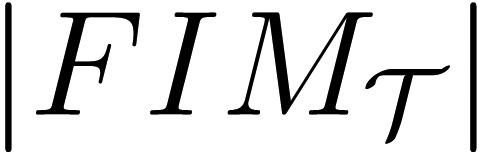
 [*t*], then *p_i_* [*t*+ 1 ] becomes the new set of sensor positions, *ζ* [*t*+ 1 ] = *ζ* [*t*] + 1, and the iteration goes back to step 1, with *p_i_* [*t*+ 1 ] = [*p_ix_* [*t*+ 1 ], *p_iy_* [*t*+ 1 ]].
5. If 
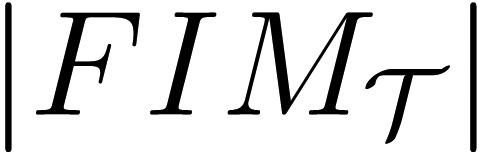
 [*t* + 1] < 
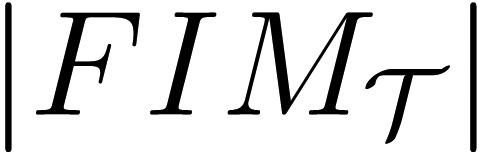
 [*t*], then there is no improvement in the convex combination of the determinants, *ζ* [*t*] = 0. The iterative algorithm stops, and *p_i_* [*t*] is considered to be the optimal configuration for the current target positions.

The above cycle is only run once if the targets are stationary. Notice the unrealistic assumption, also made in many of the publications available in this area, that the positions of the targets are known in advance. This is done to simplify the problem and to first fully understand its solution before the realistic scenario where the positions of the targets are known with uncertainty can be tackled. In this respect, see Section 7, which is largely inspired by the work in [21]. Clearly, in order for the information about the optimal configurations to be useful, one must check if the determinants of the individual FIMs for each target meet desired specifications. To evaluate how good the sensor formation is in terms of yielding accurate localization of the real targets, the determinant, 
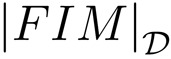
, previously introduced, is used.

In a practical situation where the targets are in motion, the sensor network must adapt its optimal configuration as the mission unfolds in three different intertwined processes:

(i) *multiple target position estimation*, albeit with a possibly large error, using the current sensor configuration and resorting to a dedicated nonlinear filter (e.g., Extended Kalman filter);
(ii) *optimal sensor configuration computation*, based on the data provided by the previous process and the algorithm described above or its modification in Section 7;
(iii) *coordinated motion control* to actually drive the moving sensors to the optimal positions determined in (ii).

The situation where the algorithm described is run during each cycle of the positioning system in (ii) is thus envisioned. Interestingly enough is also the situation where the different iterations of process (ii) can be used to yield set points for the autonomous sensor network to move to, effectively guiding them collectively to the optimal configuration that is being computed.

The advantage of using a gradient optimization method is its simplicity. As it will be seen later, based on the simulations done so far, the method has proven to be quite satisfactory. However, should there be a need for a more refined method, the sensor network positions given by the gradient algorithm can be used as initial estimates in the new method.

The rest of this section contains the results of simulations that illustrate the potential of the methodology developed for optimal sensor placement when multiple targets are involved. A large accuracy can be obtained for each of the targets, but if the mission or task would require different accuracy levels for each of the targets, then weights could be associated with them. The selection of weights would be mission-dependent, but the procedure would be very similar to that explained above.

### 6.2. Simulation Examples for Multiple Target Positioning

In what follows, some examples of optimal sensor placement for the multi-target scenario are studied to illustrate the methodology developed.

**Figure 5.**
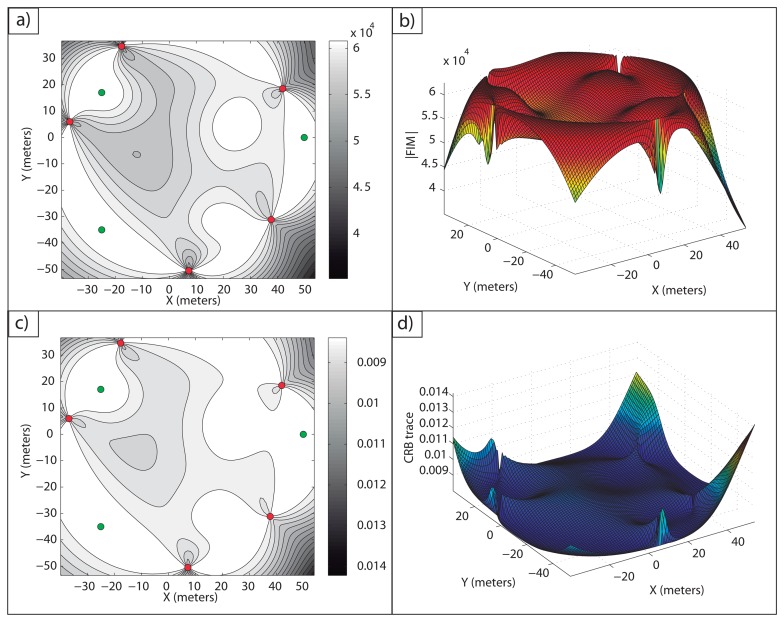
Optimal sensor formation for five sensors and three targets. In (**a**) 
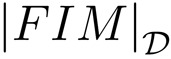
 in ℜ^2^ is shown; and in (**b**), its magnitude in 3D for 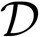. Similarly, in (**c**), 
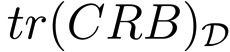
 is shown; and in (**d**), its magnitude in 3D for 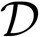.

**Example 4:** The present example deals with five sensors and three targets. With the gradient optimization algorithm described, it is possible to compute an optimal configuration with which the maximum estimation accuracy is obtained for each target, *i.e.*, Equation (47) holds for each of them. The initial guess used was a regular formation around the origin of the inertial coordinate frame with a radius of 20 meters. The target and optimal sensor positions are stated in Table 2, and in Figure 5, the optimal configuration is shown.

Figure 5a,b, shows how the theoretical maximum FIM determinant is obtained at the target positions, |*FIM*| =*n*^2^/4*σ*^4^ = 6.25·10^4^*m*^−4^. In Figure 5c,d, the theoretical minimum CRB trace is also obtained at the target positions, *tr*(*CRB*) = *σ*^2^·4*/n* = 0.08 *m*^2^.

The optimality condition in Equation (47), which holds for every target with this configuration, is not computed in this example to avoid tedious repetition of the same previous arguments, but it is easy for the reader to check, in a similar way as Example 3, that the design condition in Equation (47) is kept for each target. The computation time of the optimal solution for this particular example was 0.2808 seconds, showing again the good behaviour of the algorithm described.

**Example 5:** This example illustrates the case where there exists a large number of targets whose configuration does not allow one to obtain the maximum theoretical FIM determinant for all of them. For this purpose, seven targets and six sensors are considered. The optimal configuration is computed by resorting to the optimization algorithm previously defined and with the same initial guess used in Example 4, but in this case, Equation (47) does not hold for all targets.

The target positions and the optimal sensor formation are listed in Table 3. It is important to point out that the target positions were chosen, so that the analytical solution cannot be obtained.

**Figure 6.**
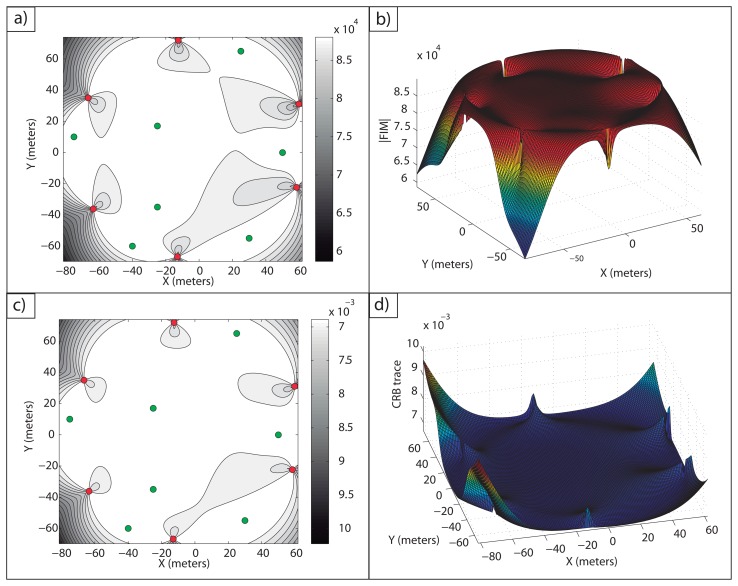
Optimal formation for six sensors and seven targets. In (**a**), 
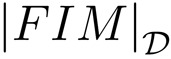
 in ℜ^2^ is shown; and in (**b**), its magnitude in 3D for 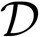. Similarly, in (**c**), 
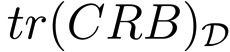
 is shown; and in (**d**), its magnitude in 3D for 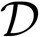.

In Figure 6a,b, it is shown how the maximum FIM determinants are obtained at the targets positions and, in Figure 6c,d, how the same occurs with the minimum CRB trace.

The FIM determinants obtained with this formation for each of the targets are stated in Table 4. It is easy to check that the FIM determinants are very close to the optimal one, |*FIM*| = *n*^2^/(*σ*^4^·4) = 6^2^/(0.1^4^·4) = 9·10^4^*m*^–4^.

The computation time in this case was 2.2308 s; so, it is clear that the time needed to obtain the optimal configuration increases for a larger number of targets. Despite this, the time required is small, and the optimal network is defined in a fast manner. Therefore, it is possible to design sensor configurations that provide the theoretical maximum FIM determinant for each target or a value very close to the maximum one.

## 7. Uncertainty in the Target Locations

In this section, the situation in which the targets to be positioned are known to lie in well-defined uncertainty regions is addressed. Inspired by the work in [21], it is assumed that the uncertainty in the target positions is described by given probability distribution functions. The objective is the maximization, by proper sensor placement, of the average value of the log determinants of the FIMs for the targets.

In what follows, *p_iξ_*; *i* = 1, 2, …, *n*; *ξ* = *x*, *y* denotes the *ξ* − *th* coordinate of sensor *i* located at position *p_i_*, $\bar{p}={\left[p_{1}^{T},\ldots p_{n}^{T}\right]}^{T}$ and $\bar{q}={\left[q_{1}^{T},\ldots q_{m}^{T}\right]}^{T}$. Further, denote by *φ*(*q̄*); *q̄* ∈ ℜ*^m^*^×2^ the probability density functions, with support, *D* ∈ ℜ^2^, that describe the uncertainty in the position of the targets in region *D*, where *D* = *D*_1_ + ⋯ + *D_m_*. With this notation, the problem of optimal sensor placement can be cast in the form of finding a vector, p̅*, such that:

(55) $${\bar{p}}^{*}=\arg\underset{\bar{p}}{\max}\int_{D}\left|\text{FIM}\left(\bar{p},\bar{q}\right)\tau \right|\cdot \varphi \left(\bar{q}\right)d\bar{q}$$

where the notation, 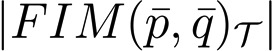, is used to clearly show the dependence of the FIM on the target and sensor locations. However, in the following, 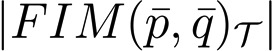 will often be denoted simply as 
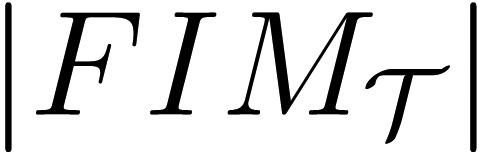
. In a real situation, *φ* (*q̅*) will depend on the type of mission carried out by the targets. Therefore, different distributions can be taken for different targets and different scenarios.

To proceed, one must compute 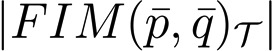 in the equation above. At this point, it is important to remark that, given the complexity of the optimal sensor placement problem at hand, the only viable solution is a numerical one.

It now remains to solve the optimization problem defined above. Conceptually, the procedure to determine the optimal sensor configuration is similar to that explained in the previous sections; that is, one must compute the derivatives of Equation (55) with respect to the sensor coordinates:

(56) $$\frac{\partial }{\partial p_{i\xi }}\int_{D}\left|\text{FIM}{\left(\bar{p},\bar{q}\right)}_{\tau }\right|\cdot \varphi \left(\bar{q}\right)d\bar{q}$$

for *i* = 1, 2,…, *n* and ξ = *x*, *y*.

To proceed with the computations, the integral and derivative operations are interchanged: the derivatives are explicitly determined first, and the integration over region *D* is performed afterwards.

The derivatives can be computed in a recursive way using Equations (50) and (51) for any number of targets. With regards to the computation of the double integral over the region *D* of interest, however, this is virtually impossible to do analytically For this reason, the integral is computed numerically with a Monte Carlo method. Finally, the solution of Equation (55) is obtained using the gradient optimization method detailed in Section 6.1. To overcome the possible occurrence of local maxima or the divergence of the algorithm, the initial guess in the iterative algorithm must be chosen with care. In the examples studied, it was found to be useful and expedient to adopt, as an initial guess, the solution for the multiple target positioning problem with the hypothetical targets placed at the center of mass of their corresponding work areas. This initial guess is obtained following the same procedure of Examples 4 and 5. It is important to stress that the solution to Equation (55) depends strongly on the probability density function adopted for the target positions, *q̅*.

### 7.1. Simulation Examples

Two different examples of multiple target positioning when the target positions are known with uncertainty are studied now. In these examples, an error model defined by *σ* = 0.1 *m* is considered, and it is only known the region in which the targets operate, instead of the target positions themselves. For the computation of the Monte Carlo integrals, 10,000 samples have been used.

**Figure 7.**
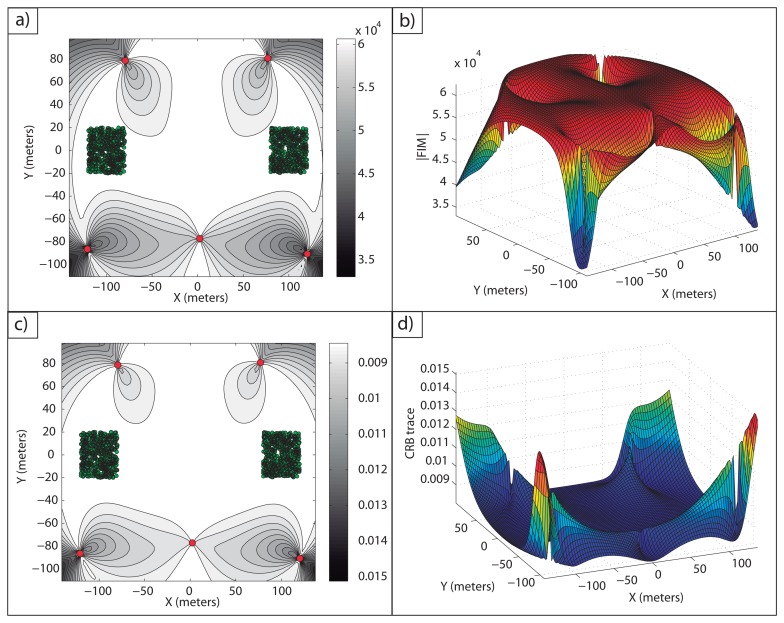
Optimal sensor placement of five sensors for two target positioning with uncertainty. In (**a**), 
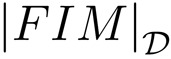
 in ℜ^2^ is shown and, in (**b**), its magnitude in 3D for 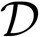. Similarly, in (**c**), 
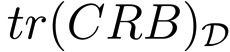
 is shown and, in (**d**), its magnitude in 3D for 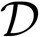. The uncertainty regions are represented by 100 samples of the total number used for the Monte Carlo computation.

**Example 6**: In this example, the scenario with two targets and five sensors is studied. The only knowledge about the target positions is that the targets operate inside a certain area; therefore, the probability distribution functions that describe the target positions are step-like distribution functions, and thus, the targets can be placed at any point inside their corresponding work areas. The areas in which the targets operate are squares of 40 × 40 *m*^2^, whose vertices are given by the points, *D*_1_ = [−120 − 20; −120 20; −80 −20; −80 20]*^T^m*, for target 1, and *D*_2_ = [80 −20; 80 20; 120 −20; 120 20]*^T^m* for target 2. The initial guess used was the optimal formation for known target positions when the targets are placed at the center of the work areas. After the optimization process referenced above, the optimal formation is defined by the sensor positions listed in Table 5 and shown in Figure 7.

It is possible to check in Figure 7a,b how a large average FIM determinant, close to the optimal one, is obtained over the work areas. In fact, the maximum and minimum determinants that this optimal formation provides inside the areas of interest are |*FIM*|*_max_* = 6.25 10^4^ m^−4^ and |*FIM*|*_min_* = 6.08·10^4^ m^−4^. Notice how the maximum determinant is the theoretical optimal one, |*FIM*|*_opt_* = n^2^/(4σ^4^) m^−4^, and how the minimum determinant is very close to this optimal value, as well, giving a large accuracy for all the points of the uncertainty regions. Moreover, the average FIM determinant inside the regions of interest is |*FIM*|*_avg_* = 6.23 m^−4^, showing a very large accuracy for the multiple target positioning task. In a similar manner, in Figure 7c,d, the CRB trace in 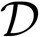 is shown, and it can be seen how inside the work areas, a small average CRB trace is obtained. The minimum and maximum CRB traces inside the areas of interest are *tr*(*CRB*)*_min_* = 0.008 *m*^2^ and *tr*(*CRB*)*_max_* = 0.0082 *m*^2^. The minimum CRB trace is again the theoretical minimum, *tr*(*CRB*)*_opt_* = 4*σ*^2^/*n m*^2^, and the maximum is also very close to this optimal value.

The computation time for the solution shown was 40.0611 s. This time is larger than in the case of known target positions, although it is still a small computation time, showing how the approach is reliable for practical mission scenarios. Thus, from this example, it is clear that it is possible to define optimal sensor configurations for which the accuracy inside the work areas is very close to the maximum accuracy that would be obtained for a single target with a known position working in isolation.

**Example 7:** The problem of three target positioning with a five sensor network is tackled now. In this example, the sensors can be placed in a wide area, similarly to the previous example. The uncertainty regions where the targets operate are square areas of 40 × 40 *m*^2^ defined by the vertices, *D*_1_ = [−100 − 20; −100 20; −60 − 20; −60 20]*^T^m*, for target 1, *D*_2_ = [100 − 20; 100 20; 60 − 20; 60 20]*^T^m* for target 2 and *D*_3_ = [−20 100; −20 140; 20 100; 20 140]*^T^m* for target 3. The only knowledge about the target positions is, again, that the targets are inside these areas, and thus, the probability distribution functions are step-like distributions. The sensor formation that maximizes the average logarithm of the FIM determinants is defined by the sensor positions shown in Table 6.

**Figure 8.**
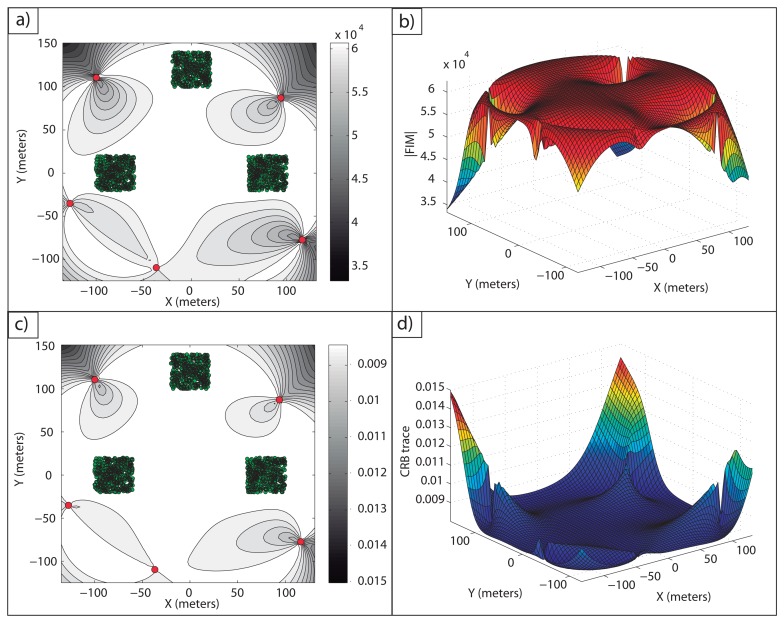
Optimal sensor placement of five sensors for three target positioning with uncertainty. In (**a**), 
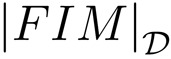
 in ℜ^2^ is shown; and in (**b**), its magnitude in 3D for 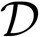. Similarly, in (**c**), 
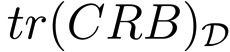
 is shown; and in (**d**), its magnitude in 3D for 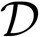. The uncertainty regions are represented by 100 samples of the total number used for the Monte Carlo computation.

Again, it is possible to notice in Figure 8 how a large accuracy is obtained inside the areas of interest.

In Figure 8a,b, the FIM determinants over *D* are shown. The maximum and minimum FIM determinants obtained inside the work areas are |*FIM*|*_max_* = 6.25 · 10^4^ m^−4^ and |*FIM*|*_min_* = 6.11 · 10^4^*m*^−4^, respectively The average value inside the regions of interest is |*FIM*|*_av_* = 6.23·10^4^*m*^−4^, providing a very large accuracy inside the work areas. Notice how the maximum FIM determinant is the theoretical one that can be obtained for a single target working in isolation, |*FIM*|*_opt_* = *n*^2^/(4*σ*^4^) m^−4^, and the minimum FIM determinant is very close to this theoretical optimal value, so that the accuracy inside the regions of interest is very close to the optimal one. Similarly, in Figure 8c,d, the trace of the CRB for each point in 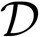 is shown. The minimum and maximum CRB traces are *tr*(*CRB*)*_min_* = 0.008 *m*^2^ and *tr*(*CRB*)*_max_* = 0.0082 *m*^2^, respectively, with the minimum value equal to the theoretical one, *tr*(*CRB*)*opt* = *4σ*^2^/*n m*^2^, and the maximum very close to it, as mentioned for the FIM determinant plots. The computation time for this example was 54.4755 s, due to the more complex situation of three uncertain areas.

Because in a practical scenario involving multiple targets, where the sensor network must adapt its configuration to the estimated positions of the targets that can also be moving, it is only required that the optimal sensor locations be computed at a rate that is much smaller than the rate at which control systems are run in real time, the commented computation times show that the algorithm can be easily implemented in practice and yields good accuracy. Moreover, adding parallelism to the computations will further reduce this computation time.

Therefore, with the methodology developed, it is possible to define optimal sensor configurations to localize multiple targets, whose positions are known with uncertainty, with very large accuracy.

## 8. Conclusions

The problem of optimal sensor placement for multiple target positioning with range-only measurements in 2D scenarios has been studied. This problem is of key importance in multiple application scenarios in which a variable number of targets must be localized with the largest possible accuracy. It has been shown that in many situations of interest, it is possible to define an analytical solution for which the maximum FIM determinant is obtained for each of the targets involved in the task. Despite this, there exist some complex target formations that do not allow for analytical solutions. For this case, convex optimization tools have been employed to determine the optimal sensor configurations. Moreover, the numerical solutions determined with the optimization algorithm provide accuracies for each of the targets very close to that obtained in the ideal case for a single target working in isolation. Some illustrative examples have been developed to show these issues.

The previous results were extended to the more realistic problem, where the target positions are known with uncertainty. This uncertainty can be defined by any probabilistic distribution function, and the type of function used conditions the optimal sensor formation. An optimization method similar to that previously defined was used to determine the optimal sensor configurations. The main problem to overcome was the resolution of the integrals of the gradient equations, to determine the necessary gradients to increase the average FIM determinant over the work areas in the optimization algorithm. These integrals were solved numerically by a Monte Carlo method, because of the impossibility of solving them analytically. It has been shown through several examples how it is possible to compute optimal formations for which the accuracy obtained inside the uncertainty areas is very close to the ideal one and how it is possible to localize multiple targets with significant accuracy, no matter the configuration of the targets.

Future work will aim to: (i) extend and apply the methodology developed to a real multiple vehicle mission scenario and (ii) study the performance of the algorithms for the optimal sensor configuration computation developed herein, together with selected algorithms for target tracking and cooperative sensor motion control.

## Figures and Tables

**Table 1.** Target positions and optimal sensor positions.

	***q*_1_**	***q*_2_**	***p*_1_**	***p*_2_**	***p*_3_**	***p*_4_**
{*x_I_*} − *coordinate* (*m*)	20	−20	−26.39	−26.39	26.39	26.39
{*y_I_*} − *coordinate* (*m*)	0	0	−17.22	17.22	−17.22	17.22

**Table 2.** Target positions and optimal sensor positions.

	***q*_1_**	***q*_2_**	***q*_3_**	***p*_1_**	***p*_2_**	***p*_3_**	***p*_4_**	***p*_5_**
{*x_I_*} − *coordinate* (*m*)	−25	−25	50	42.00	−17.68	−36.82	7.23	37.65
{*y_I_*} − *coordinate* (*m*)	17	−35	0	18.48	34.57	5.99	−50.50	−31.09

**Table 3.** Target positions and optimal sensor positions.

	***q*_1_**	***q*_2_**	***q*_2_**	***q*_4_**	***q*_5_**	***q*_6_**	***q*_7_**
{*x_I_*} – *coord*. (*m*)	−25	−25	50	−75	25	30	−40
{*y_I_*} – *coord*. (*m*)	17	−35	0	10	65	−55	−60

	***p*_1_**	***p*_2_**	***p*_3_**	***p*_4_**	***p*_5_**	***p*_6_**	

{*x_I_*} – *coord*. (*m*)	59.85	−12.51	−66.44	−63.50	−13.03	58.41	
{*y_I_*} – *coord*. (*m*)	31.09	71.91	35.03	−36.12	−66.76	− 22.34	

**Table 4.** Fisher Information Matrix (FIM) determinants for each of the targets.

	***q*_1_**	***q*_2_**	***q*_3_**	***q*_4_**	***q*_5_**	***q*_6_**	***q*_7_**
|*FIM*| (10^4^*m*^−4^)	8.9964	8.9999	8.996	8.9997	8.9974	8.9916	8.9974

**Table 5.** Optimal sensor positions.

	***p*_1_**	***p*_2_**	***p*_3_**	***p*_4_**	***p*_5_**
{*x_I_*} – *coord*.(*m*)	75.90	−74.92	−109.96	6.82	114.51
{*y_I_*} – *coord*. (*m*)	100.96	96.67	−92.70	−51.49	−97.39

**Table 6.** Optimal sensor positions.

	***p*_1_**	***p*_2_**	***p*_3_**	***p*_4_**	***p*_5_**
{*x_I_*} – *coord*. (*m*)	116.32	93.83	−99.74	−127.45	−36.87
{*y_I_*} – *coord*. (*m*)	−77.46	87.18	110.65	−35.18	−109.61
